# Clinical Genetic Testing for the Cardiomyopathies and Arrhythmias: A Systematic Framework for Establishing Clinical Validity and Addressing Genotypic and Phenotypic Heterogeneity

**DOI:** 10.3389/fcvm.2016.00020

**Published:** 2016-06-27

**Authors:** John Garcia, Jackie Tahiliani, Nicole Marie Johnson, Sienna Aguilar, Daniel Beltran, Amy Daly, Emily Decker, Eden Haverfield, Blanca Herrera, Laura Murillo, Keith Nykamp, Scott Topper

**Affiliations:** ^1^Invitae Corporation, San Francisco, CA, USA

**Keywords:** genetic testing, cardiomyopathies, arrhythmias, ARVD/C, curation

## Abstract

Advances in DNA sequencing have made large, diagnostic gene panels affordable and efficient. Broad adoption of such panels has begun to deliver on the promises of personalized medicine, but has also brought new challenges such as the presence of unexpected results, or results of uncertain clinical significance. Genetic analysis of inherited cardiac conditions is particularly challenging due to the extensive genetic heterogeneity underlying cardiac phenotypes, and the overlapping, variable, and incompletely penetrant nature of their clinical presentations. The design of effective diagnostic tests and the effective use of the results depend on a clear understanding of the relationship between each gene and each considered condition. To address these issues, we developed simple, systematic approaches to three fundamental challenges: (1) evaluating the strength of the evidence suggesting that a particular condition is caused by pathogenic variants in a particular gene, (2) evaluating whether unusual genotype/phenotype observations represent a plausible expansion of clinical phenotype associated with a gene, and (3) establishing a molecular diagnostic strategy to capture overlapping clinical presentations. These approaches focus on the systematic evaluation of the pathogenicity of variants identified in clinically affected individuals, and the natural history of disease in those individuals. Here, we applied these approaches to the evaluation of more than 100 genes reported to be associated with inherited cardiomyopathies and arrhythmias including hypertrophic cardiomyopathy, dilated cardiomyopathy, arrhythmogenic right ventricular dysplasia or cardiomyopathy, long QT syndrome, short QT syndrome, Brugada, and catecholaminergic polymorphic ventricular tachycardia, and to a set of related syndromes such as Noonan Syndrome and Fabry disease. These approaches provide a framework for delivering meaningful and accurate genetic test results to individuals with hereditary cardiac conditions.

## Introduction

The dramatic reduction in the cost of DNA sequencing, combined with advances in the understanding of the genetic and phenotypic heterogeneity of cardiac conditions, has led to the adoption of large panels of genes as a cost-effective clinical tool to establish a molecular diagnosis in affected individuals. But, although the economics and the potential yield of such tests have improved, the fundamental principles of diagnostic testing – analytic validity, clinical validity, and clinical utility – have not.

Genetic diagnosis of inherited cardiac conditions is especially challenging due to the extensive genetic heterogeneity and the overlapping, variable, and incompletely penetrant nature of the clinical presentations. The design of effective diagnostic tests and the effective use of the results of such tests depend on a clear understanding of the relationship between each gene and each considered condition, and a clear understanding of the extent of the overlap in clinical presentation.

As part of an effort to develop a scalable framework for developing, launching, and supporting diagnostic genetic tests across a broad range of clinical areas, we set out to create general methods for establishing the *clinical validity* of a gene, and for designing focused and clinically useful panel tests.

Establishing the clinical validity of a multi-gene panel depends on an accurate and detailed understanding of the clinical validity of each included gene. While clinical laboratories have been implicitly making assessments regarding clinical validity for years, there has been a lack of clarity about evidentiary requirements for establishing clinical validity and, especially for rare, multigenic conditions, accessible data and methods to be applied in reaching this conclusion.

Within clinical genetic diagnostics, clinical validity “measures the accuracy with which a test identifies a person with the clinical condition in question” (1–3) and depends on such quantitative measures as sensitivity, specificity, positive predictive value and negative predictive value. While there are disagreements about quantitative thresholds for these measures, this is clear: a test that cannot return a positive result has no sensitivity and no positive predictive value, and cannot be considered clinically valid. Conceptually, clinical validity can be understood as a proven, causal connection between a gene and a human disease.

For diagnostic testing, the question of clinical validity should be thought of as only the first requirement. Once answered positively, it opens onto a series of more detailed considerations that also need evaluation. If it has been proven that the gene causes human disease (i.e., if clinical validity has been established) then one can reasonably ask: which disease(s)? How certain are we that we understand the boundaries of the phenotypic heterogeneity that can derive from this gene? What is the yield in different populations? Which specific variants are causal? How certain are we that we completely understand the molecular mechanisms? If, however, it has not yet been proven that a gene causes disease, then questions of expressivity and mechanism of disease are clouded by this more fundamental uncertainty.

This paper aims to (1) propose a method for establishing clinical validity of a gene, (2) propose a method for grouping genes together into meaningful panel tests, and (3) apply those methods to evaluate a set of cardiac genes and conditions. We also describe the kinds of specific results that may be expected from clinical testing of different classes of genes, and the appropriate use of those results in clinical care.

## Materials and Methods

When we first began curating gene–condition relationships, we established a working group to develop a framework for evaluating and documenting relevant evidence and our conclusions about that evidence. The working group consisted of lab directors, genetic counselors, and scientists with experience from a diversity of diagnostic and research labs. We first discussed and compared methods used in those environments, and quickly came to the conclusion that, while different approaches generally considered the same *types* of evidence (linkage studies, animal, cellular, and molecular models, observations of variants in affected individuals and pedigrees), there was little consensus about how to rigorously and reproducibly synthesize that evidence. In fact, in many cases that synthesis was not supported by an established method, but rather left to the professional judgment of a single individual.

As a starting point, the working group established a simple point-based framework that focused on a comprehensive cataloging of the relevant experimental and observational evidence, judging the strength of that evidence, and requiring that multiple pieces of evidence were present. We then applied that preliminary framework to the curation of a set of genes putatively involved in an array of hereditary cancers. While the framework was generally effective, we found that progress was slow, that much of the research we were doing had little long-term utility in the context of a diagnostic lab, and that there were regularly cases that led to a kind of logical conflict: genes supported by extensive and generally convincing research, where we were still unable to deliver positive results without additional clinical observations or experimental data.

We therefore re-convened the working group to reconsider the framework, with the following goals: (1) clearly define the purpose of the curation research effort, (2) identify the specific information that directly supported those aims, and (3) develop a reproducible and auditable approach to meet those aims. The group identified and discussed particular cases that led to disagreements or inconsistency when using the previous approach, and worked through a series of thought experiments to elucidate edge cases. Through this process, the logical necessity of harmonizing variant evaluation and gene clinical validity evaluation emerged. As described below, the method that was developed simply defers the general question of the clinical validity of a gene to the specific question of the pathogenicity of clinically observed variants.

With this framework in place, we then applied the method to the set of genes suggested to cause hereditary cardiac conditions. For each considered gene, we used the Human Gene Mutation Database (HGMD) to provide a list of published, clinically observed variants. The presence of a variant in the HGMD database does not necessarily indicate pathogenicity, and variants were independently researched and interpreted using an implementation of the ACMG variant interpretation guidelines. In the case where none of the variants listed in HGMD were determined to be convincingly pathogenic, a literature search for more recent case reports of other clinically observed variants was performed.

## Results

### Establishing the Clinical Validity of Gene–Condition Relationships

#### Method for Establishing the Clinical Validity of a Particular Gene

The approach described here addresses the question of distinguishing between genes that have been proven to cause human disease and genes that currently only have preliminary evidence suggesting an association. It depends on a simple insight: an accurate methodology for evaluating *variant pathogenicity* must provide results that are consistent with an accurate methodology for evaluating the *clinical validity* of the gene. If the methodologies provide inconsistent results, one of the methodologies must be delivering an incorrect conclusion. This dependency suggests that the approaches can and should be harmonized.

This dependency can be demonstrated with a logical argument:

- Clinical validity of a gene is established when we know that the gene causes human disease.
- A gene causes human disease because at least one variant in the gene causes human disease.
- Therefore, clinical validity of a gene is established when we know of at least one variant in the gene that causes human disease.
- If the variant classification schema is robust, we know that a variant causes disease only when there is sufficient evidence to classify the variant as pathogenic.
- Therefore:?
  ◦ If there is sufficient evidence to classify a variant as pathogenic, then we know that the variant causes disease, we know that the gene causes disease, and we know that a clinical test of the gene can be clinically valid.
- Conversely:
  ◦ If there is NOT sufficient evidence to classify any clinically observed variants as pathogenic (i.e., if ALL clinically observed variants must be classified as VUS), then we do not know of a variant in the gene that has been proven to cause disease in humans, we can not be certain that the gene causes disease, and we do not know that a test of the gene would be clinically valid.

This argument reduces the question of evaluating the strength of the evidence supporting a causal *gene–condition* relationship to the more tractable question of the formal classification of clinically observed *variants*.

This approach depends on a robust and rigorous variant classification method, such as a careful implementation of the American College of Medical Genetics (ACMG) variant classification guidelines. These guidelines classify observations about the consequence and context of a variant into one of a series of “evidence types.” Each evidence type contributes a predetermined amount to the argument that a variant is benign or pathogenic, and thresholds are suggested for the amount of evidence required to reach a certain classification. Admissible evidence may include family segregation data, observations in multiple, unrelated, clinically affected individuals, absence in healthy controls, animal models demonstrating recapitulation of a human disease phenotype, and functional data demonstrating an aberrant effect on the protein or transcript. The conclusion that a variant is pathogenic generally requires more than one type of strong evidence consistent with pathogenicity, and generally requires the observation of the variant in multiple, unrelated, similarly affected individuals.

The number of different pathogenic variants a gene harbors is not relevant to the general question of clinical validity. In fact there are many cases, especially in genes with gain-of-function disease mechanism, where all known incidences of the disease are caused by the same, specific pathogenic variant. In these cases, the clinical validity of the gene test is still established by the determination that that single variant is pathogenic.

One possible objection is that the assessment of a variant’s pathogenicity depends on already knowing the strength of the gene–condition relationship. We would argue that a careful application of the variant classification framework already takes the relevant uncertainties into account. The ACMG framework provides a series of cautions about using information of tangential relevance: for example, case reports from individuals with possibly unrelated presentations, or assumptions about molecular mechanism. The power of case reports should be modulated based on the relevance and specificity of the presenting phenotype. Before the gene has been well established as a cause of disease, any case report should be treated with this caution. The significance of the effect of the change on the RNA or protein should be modulated based on the understanding of the molecular mechanism of disease. Before the gene has been well established as a cause of disease, all sequence observations should be treated with this caution. If these cautions are respected, it will be impossible to conclude that a variant is pathogenic without substantial evidence of multiple types supporting the conclusion.

If there is a clinically observed variant that can be classified as pathogenic, then it has been proven that the gene causes human disease.

#### Gene–Condition Strength Terminology

We describe the strength of the evidence supporting a possible relationship between a gene and a particular condition as either “strong,” “suggested,” or “emerging.”

- “Strong” is used in cases where there exists at least one clinically observed variant supported by sufficient evidence to classify that variant as pathogenic. “Strong” indicates that the relationship has been proven.
- “Suggested” is used in cases where some preliminary evidence exists suggesting a causal relationship, but the relationship has not yet been formally proven.
- “Emerging” is used to describe a growing suspicion that a *specific* condition is caused by a gene that has already been proven to cause disease.

If a gene has at least one “strong” relationship, then clinical validity for that gene has been established. If a gene has no “strong” relationships, then clinical validity has not been established, and the gene remains a “Gene of Uncertain Significance.”

#### Establishing Specificity in the Associated Clinical Phenotype

It is important to be as specific as possible with regard to the condition(s) associated with a gene. However in some cases we recognize that, while it is clear that the gene causes disease, there are too few case reports to derive any confidence about *which* disease, or if that disease matches neatly with any known and established clinical entities.

We address this question by a two-step process. We first establish that the gene causes disease by considering its relationship to a generic entity referred to as “GENE-related conditions.” We then consider if that generic entity can be refined into one or more specific conditions.

Our approach to this question relies on a heuristic. In order to consider the relationship between a gene and a specific condition as “strong,” we require the observation of a pathogenic variant in three unrelated individuals who manifest the specific condition. If only one or two case reports describing individuals with pathogenic variants and manifesting a specific condition are available, we consider the specific gene–condition relationship to be “emerging.” The decision to require three individuals is not a statistical assessment, but is meant as a simple hedge against coincidences of individual expressivity or complex individual genotype; a non-classical clinical presentation in an affected individual may simply reflect an expansion of disease presentation or a modification of the presentation due to other genetic or external modifiers.

A gene can be well established as the cause of one specific condition, and also purported to cause an additional condition. In some cases, the conditions are distinct enough to be thought of as separate entities, and in some cases, the second condition should be thought of as a phenotypic expansion of the gene-specific, clinical manifestation. This distinction can be somewhat subjective. In general, if individuals with pathogenic variants and both phenotypes are reported, we consider this to be evidence in support of the idea that the phenotype associated with that gene is complex, rather than the idea that the gene causes two distinct conditions.

This is of special importance in cardiac genetics where some distinct conditions exist, where common mechanisms may cause one clinical condition to progress to presenting features of a second, and where there is extensive clinical overlap between some closely related conditions. Furthermore, because some cardiac conditions display reduced penetrance and/or later onset, the simple fact of observing a pathogenic variant in a single individual with an unexpected phenotype is not sufficient to establish a relationship between this variant and the carrier’s condition.

### Evaluation of Cardiac Genes: Examples and Summary

For each gene purported to be associated with a cardiac condition, we evaluated the evidence supporting the pathogenicity of the published, clinically observed variants. The full conclusions of our assessments of the genes associated with arrhythmias, cardiomyopathies, and the related syndromes are presented in Tables 1 and 2, and detailed examples of Strong, Suggested, and Emerging relationships are described below.

**Table 1:** **Gene–condition strengths for selected cardiomyopathies**.

	HCM	DCM	ARVD/C	LVNC	Overlapping cardiomyopathy syndromes
*MYBPC3*	Strong	Strong		Suggested	

*MYH7*	Strong	Strong		Strong	Strong: Laing distal myopathy

*PLN*	Strong	Strong	Strong	Emerging	

*TNNC1*	Strong	Strong			

*TNNT2*	Strong	Strong		Strong	

*TPM1*	Strong	Strong		Suggested	

*TNNI3*	Strong	Strong		Suggested	

*TTN*		Strong			Strong: titinopathies

*LMNA*		Strong	Suggested	Suggested	Strong: laminopathies

*DSP*		Strong	Strong		Strong: Carvajal syndrome

*RBM20*		Strong	Suggested		

*VCL*	Emerging	Strong			

*TAZ*		Strong		Strong	Strong: Barth syndrome

*DSG2*		Strong	Strong		

*DES*		Strong	Strong		Strong: desminopathy

*BAG3*		Strong			Strong: myofibrillar myopathy

*SCN5A*		Strong	Strong	Suggested	

*DMD*		Strong			Strong: Duchenne muscular dystrophy

*RAF1*		Strong			

*ACTC1*	Strong	Emerging		Strong	

*ACTN2*	Strong	Emerging	Emerging		

*CSRP3*	Strong	Suggested			

*FHL1*	Strong				Strong: Emery–Dreifuss muscular dystrophy

*GLA*					Strong: Fabry disease

*MYL2*	Strong				

*MYL3*	Strong				

*PRKAG2*	Strong				Strong: glycogen storage disease

*HCN4*				Strong	

*RYR2*		Emerging	Strong	Strong	

*LAMP2*		Emerging			Strong: Danon disease

*TTR*					Strong: transthyretin amyloidosis

*ELAC2*					Strong: combined oxidative phosphorylation deficiency

*MTO1*					Strong: combined oxidative phosphorylation deficiency

*CAV3*	Suggested				Strong: caveolinopathies

*ALMS1*					Strong: Alstrom syndrome

*EMD*		Emerging			Strong: Emery–Dreifuss muscular dystrophy

*FKRP*					Strong: muscular dystrophy

*FKTN*					Strong: muscular dystrophy

*JUP*		Suggested	Strong		Strong: Naxos disease

*SDHA*					Strong: mitochondrial complex II deficiency

*TMEM43*			Strong		Suggested: Emery–Dreifuss muscular dystrophy

*DSC2*		Suggested	Strong		Strong: RVC with palmoplantar keratoderma and wooly hair

*PKP2*		Suggested	Strong		

*TCAP*	Suggested	Suggested			Strong: limb girdle muscular dystrophy

*ABCC9*		Suggested			Strong: Cantu syndrome

*SGCD*		Suggested			Strong: limb girdle muscular dystrophy

*This table presents assessments of the strength of each gene–condition associations across the cardiomyopathies. Specific references for each cell in this table are available in the Supplementary Material*.

*The following genes have only “suggested” relationships to cardiac conditions, and are therefore classified as preliminary evidence genes: LDB3, ANKRD1, PDLIM3, MYPN, NEXN, CALR3, JPH2, MYLK2, MYOM1, MYOZ2, PRDM16, CRYAB, CTF1, FHL2, GATA6, GATAD1, ILK, LAMA4, NEBL, NPPA, TMPO, TXNRD2, DTNA, CTNNA3*.

**Table 2:** **Gene–condition strengths for selected arrhythmias**.

	Long QT syndrome	Short QT syndrome	Brugada syndrome	CPVT	Overlapping arrhythmia syndrome
*KCNH2*	Strong	Strong	Suggested		

*KCNQ1*	Strong	Emerging			Strong: Jervell Lange-Nielsen syndrome

*SCN5A*	Strong		Strong		

*CASQ2*				Strong	

*RYR2*				Strong	

*CAV3*	Strong				

*KCNE1*	Strong				Strong: Jervell Lange-Nielsen syndrome

*KCNE2*	Strong				

*CALM1*	Strong			Strong	

*CALM2*	Strong			Strong	

*CALM3*	Strong			Strong	

*TRDN*	Strong			Strong	

*KCNJ2*	Emerging	Strong		Emerging	Strong: Anderson-Tawil

*CACNB2*		Strong	Strong		

*GPD1L*			Strong		

*CACNA1C*	Strong	Suggested	Suggested		Strong: Timothy syndrome

*This table presents assessments of the strength of each gene–condition associations across the arrhythmias. Specific references for each cell in this table are available in the supplemental materials*.

*The following genes have only “suggested” relationships to cardiac conditions, and are therefore classified as preliminary evidence genes: SCN4B, SNTA1, TRPM4, KCNE3, KCNE5, RANGRF, SLMAP, KCNJ8, SCN3B, SCN2B, and SCN10A*.

*CPVT, catecholaminergic polymorphic ventricular tachycardia*.

#### Establishing a Single Condition as “Strong”

The *MYH7* gene has long been understood to be a cause of Hypertrophic Cardiomyopathy (HCM), a relationship which is clearly illustrated by the well-known, pathogenic p.Arg403Gln variant. This variant is absent from control populations, but has been shown to strongly segregate with HCM in four families with an overall LOD score of 3.4 (4–7). In addition, experimental studies have demonstrated that this change leads to defective ATPase activity and significantly alters actin motility (8–13). Furthermore, this variant has been shown to cause HCM in both transgenic mouse and rabbit models (14, 15). The clinical, functional, population, and animal data clearly establish this variant as pathogenic, and an abundance of individuals with this variant and a classic HCM phenotype have been reported. This pathogenic variant in *MYH7* causes HCM, and the link between *MYH7* and HCM is therefore established.

#### Multiple “Strong” Conditions Caused by the Same Gene

*KCNQ1*, the potassium voltage-gated channel, is an example of a gene that causes two clinically distinct conditions: Jervell and Lange-Nielsen Syndrome (JNLS), and long QT syndrome (LQTS).

Jervell and Lange-Nielsen Syndrome is an autosomal recessive, multisystem disorder characterized by congenital profound bilateral sensorineural hearing loss and prolonged QT interval at a young age. Onset of cardiac symptoms typically occurs in childhood, and arrhythmia due to JLNS may result in recurrent syncope, seizure-like activity, or sudden cardiac arrest/death. In addition to congenital hearing loss and cardiac symptoms, some individuals with JLNS have also been found to have anemia and elevated levels of the hormone gastrin.

Although a range of variants can be pathogenic, a common pathogenic JLNS variant is p.Arg518*, a founder mutation in the Swedish population. It has been observed in the homozygous state in a number of JLNS patients, and as a compound heterozygote with other truncating variants (16–18).

In addition, heterozygous carriers of pathogenic variants are affected by LQTS of varying severity. LQTS is characterized by a prolonged QTc interval on electrocardiogram (ECG/EKG) and cardiac arrhythmia, such as torsade de pointes, that may result in recurrent syncope, seizure-like activity, and sudden cardiac arrest/death (19, 20). Although mild hearing loss can sometimes be an associated symptom of LQTS, it is a recognizably distinct clinical entity from JLNS. In one study, 12 heterozygous carriers of the pathogenic p.Arg518* variant were demonstrated to have prolonged QT segments and normal hearing (18).

This series of case studies of individuals with variants that are known to be pathogenic, and who are affected by LQTS and *not* JLNS, establishes the relationship between *KCNQ1* and LQTS.

#### “Strong” First Condition and “Emerging” Second Condition

In addition to LQTS and JLNS, there is some emerging evidence that certain variants in *KCNQ1* also cause Short QT Syndrome (SQTS). Short QT syndrome is characterized by a shortened QTc interval on electrocardiogram and cardiac arrhythmias that may result in syncope, seizure-like activity, and/or sudden cardiac arrest/death (21, 22). To date, there are two relevant case reports: (1) a 70-year-old male has been observed with SQT and a p.Val307Leu variant. While there is functional evidence that p.Val307Leu could contribute to a gain-of-function phenotype (23), this variant would still be formally classified as a VUS until additional case reports or segregation data became available. (2) An infant with severe fetal bradycardia, irregular rhythm, and short QT who has a *de novo* p.Val141Met variant. In this case also, there is some functional evidence that this mutation has a gain-of-function effect (24). The *de novo* observation contributes strongly to this variant’s pathogenic classification.

At this time, there is one report of an individual with a pathogenic variant in *KCNQ1* and a severe short QT/arrhythmogenic phenotype. It is quite likely that certain gain-of-function mutations in *KCNQ1* cause short QT; however, it is also possible that these variants are coincidental observations in individuals whose true causative variant remains undiscovered. Until additional case reports come to light, we classify the relationship between *KCNQ1* and short QT syndrome as “emerging.”

#### Single Condition Example: “Suggested”

*SCN10A* may be a gene that causes Brugada syndrome, although this has not yet been proven. While *SCN5A* is the primary cause of Brugada syndrome, rare *SCN10A* variants have been found in about 16% of *SCN5A*-negative Brugada patients. All told, there have been around 30 missense changes observed in Brugada patients (25, 26); however, a detailed evaluation of the underlying evidence regarding each of these variants leads us to conclude that every one of these variants should be classified as VUS. Most of these variants are supported by little evidence beyond an observation in an individual, absence in the general population, and computational predictors. There are two reported variants that have been explored more thoroughly, p.Arg14Leu and p.Arg1268Gln. These variants have each been observed in four individuals with Brugada signs, and experimental evidence seems to demonstrate that in an *in vitro* co-expression model, introducing these variants into *SCN10A* leads to a significant reduction in *SCN5A* current (26). However, these variants are also relatively common in the general population, with hundreds of observations in ExAC. Taken together, and in the absence of compelling segregation data, even these two variants must remain classified as VUSes.

At this point, there exist no clinically observed variants in *SCN10A* that can be classified as pathogenic, and therefore we cannot be certain that pathogenic variants in *SCN10A* cause Brugada syndrome. For this reason, we classify the relationship between *SCN10A* and Brugada as “suggested.”

#### Syndromic Genes and Isolated Phenotypes

Pathogenic variants in genes that are primarily associated with syndromes can sometimes manifest clinically as isolated cardiac conditions. This can be because other symptoms have not yet developed, because other symptoms are subtly present and have escaped notice, or because the gene truly also causes the isolated phenotype. How should we think about the range of conditions caused by mutations in these genes?

For example, do some pathogenic variants in *DMD* cause isolated dilated cardiomyopathy (DCM)? The evidence that they may includes: (1) in one large family, isolated DCM in the absence of Duchenne or Becker muscular dystrophy mapped to the *DMD* gene (27), (2) a collection of case studies identified classically pathogenic *DMD* variants (exonic deletions or splice variants) in individuals with DCM and without additional features associated with muscular dystrophy (28–30), (3) a study which evaluated the *DMD* gene (called DYS in this paper) in a series of 436 male patients diagnosed with isolated DCM, the authors identified pathogenic deletions or splice variants in 34 patients (31). Upon closer inspection, many of these individuals with “isolated” DCM had elevated serum creatine phosphokinase and/or mild skeletal myopathy. However, there were six individuals with classically pathogenic *DMD* variants who did not have any signs of latent or undiagnosed muscular dystrophy.

There are, therefore, a collection of individuals with isolated DCM whose phenotypes can clearly be explained by identified, pathogenic variants in *DMD*. We would argue that this series of patients establishes the relationship between *DMD* and isolated DCM. We would also suggest, however, that this is largely a semantic distinction. One can say, “Pathogenic variants in *DMD* can, in some cases, lead to isolated DCM” or one can say “Pathogenic variants in *DMD* cause Becker syndrome, which in some cases can present as (and not progress beyond) isolated DCM” and these amount to much the same thing in practice. A patient who has what is apparently isolated DCM should be evaluated for potential pathogenic variants in the *DMD* gene, as such a variant may be the cause of this patient’s condition. Likewise, a patient with a pathogenic variant in *DMD* should be carefully examined and monitored for other symptoms of Becker muscular dystrophy as additional symptoms may be subtle or may appear with a later onset.

*FHL1* presents another example of this logic. *FHL1* is a well established, “strong” cause of Emery Dreifuss muscular dystrophy (EDMD) and may also cause isolated HCM. Some EDMD patients develop HCM (32), and there are many reports of patients with pathogenic variants in *FHL1* who present with isolated HCM and who have no other symptoms of EDMD. These include: (1) a small pedigree of three individuals with isolated HCM that segregates with an *FHL1* truncation variant, (2) an unrelated individual with isolated HCM and an apparently *de novo* frameshift variant (33), and (3) a three generation pedigree manifesting HCM that segregates with different truncating *FHL1* variants (34). This series of individuals with pathogenic variants in *FHL1*, with a clinical diagnosis of HCM but with no evidence of EDMD, establishes the relationship between *FHL1* and isolated HCM. However, there is no clear genotype/phenotype correlation distinguishing variants that cause EDMD from variants that cause isolated HCM, and the differing manifestations may be due to other genetic or environmental factors specific to these individuals or families. Patients with isolated HCM should be evaluated for variants in the *FHL1* gene, and patients with pathogenic *FHL1* variants should be carefully examined for features of EDMD.

### Panel Design and Clinical Overlap among Cardiac Conditions

Conventional cardiac evaluations may not accurately determine an individual’s true, underlying diagnosis. For example, left ventricular hypertrophy observed on an echocardiographic evaluation is typically associated with isolated HCM but may also be the primary presenting feature of an unrecognized syndromic condition such as Noonan syndrome or Fabry disease (35). “End-stage” HCM is characterized by left ventricular dilation, and isolated echocardiogram results can easily lead to a misdiagnosis of primary DCM (36). Ventricular arrhythmia, conduction disease, cardiac arrest or unexplained syncope, in the absence of secondary causes, could either represent a primary inherited arrhythmia syndrome or the early clinical presentation of an arrhythmogenic cardiomyopathy (37–41). Clinicians and professional organizations have recognized the importance of comprehensive genetic testing to aid in the diagnosis of cardiac conditions (42), and panel design should address these issues of overlapping and misleading clinical presentation.

We propose that a comprehensive panel test designed for the molecular diagnosis of a particular condition should include the following classes of genes:

- Genes that have been conclusively proven to cause the condition in question.
- Genes suspected but not yet proven to cause the condition in question.
- Genes that have been conclusively proven to cause a condition within the clinical differential. This category should include genes that cause a condition that can progress into the condition in question, genes that cause a condition that can be confused with the condition in question, and genes that cause a syndrome that include the condition in question as a primary feature.

We suggest that the clinical validity of a panel is established when that panel includes a set of genes that account for a substantial proportion of the genetic causes of the disease in question. Conversely, a panel is NOT valid if it omits certain genes that account for a substantial proportion of the known genetic risk. A clinically valid panel may also include genes for which some preliminary evidence of clinical validity exists (“preliminary evidence genes”).

A panel test for HCM should include, therefore, genes proven or suspected to cause isolated HCM, and genes *proven* to cause conditions that can present with HCM as a primary feature, such as Fabry disease.

Likewise, a panel for DCM should include genes proven or suspected to cause isolated DCM, genes proven to cause HCM (because HCM can progress to, and be observed as, DCM), and genes proven to cause arrhythmogenic right ventricular dysplasia or cardiomyopathy (ARVD/C, because ARVD/C can present as DCM).

A selected mapping of clinically presenting features to their potential underlying clinical conditions is presented in Table 3. While this mapping is not meant to be comprehensive, it is intended to illustrate some of the common discrepancies and overlaps.

**Table 3:** **Clinical overlap of selected inherited arrhythmias and cardiomyopathies**.

	…may actually represent a true case of:											

		HCM	DCM	ARVD/C	Long QT	Short QT	Brugada	CPVT	Unspecified arrhythmia	Noonan syndrome/RASopathy	Fabry disease^a^	DMD/Becker^a^
A clinical presentation of…
	HCM	✓								✓	✓	

	DCM	✓	✓	✓								✓

	Unspecified cardiomyopathy	✓	✓	✓						✓	✓	✓

	ARVD/C		✓	✓			✓	✓	✓			

	Unspecified arrhythmia	✓	✓	✓	✓	✓	✓	✓	✓			

*Specific cardiac presentations can reflect a wide range of underlying conditions. Understanding the spectrum of conditions that can present with particular features is an important first consideration in the planning of diagnostic panels. This table presents some of these relationships*.

*^a^Noonan, Fabry and DMD/Becker are included as representative examples only. Other overlapping syndromic conditions exist but are not represented in this table [adapted from Pagon et al. (44)]*.

## Discussion

The three pillars of effective diagnostic medicine are analytic validity, clinical validity, and clinical utility. Establishing the clinical validity of a multi-gene panel depends on an accurate and detailed understanding of the strength of the evidence that establishes a causal relationship between the included genes and human disease. We have established methods to establish gene-level clinical validity, to construct meaningful panel tests, and have applied these methods to a set of cardiac gene–condition pairs.

Before the advent of exome sequencing, gene–conditions associations were traditionally established through the use of gene-mapping techniques. This approach required the ascertainment of multiple, large affected families to provide sufficient power to establish linkage to relatively small genomic regions. Genes within the identified regions were then analyzed further for possibly causal variants, or for functional or biological relevance. This was an effective strategy when the cost of expansively sequencing an individual was prohibitive, but was limited in that it relied on the availability of large pedigrees or multiple pedigrees that shared the same underlying genetic cause. Sufficient families are generally only available in the cases where a single gene explains a substantial number of cases of a particular condition.

As the cost of sequencing has come down, it has become feasible to bypass the process of narrowing the genomic search space, and to move directly to the search for causal variants. This has allowed the clinical research community to take greater advantage of isolated unrelated individuals and small pedigrees to generate meaningful genetic hypotheses. The increased accessibility of exome sequencing, for example, has led to an explosion of hypotheses about gene–condition relationships. The consequence is that we have a greater appreciation of the specific genetic diversity underlying many conditions, but also that the amount of data available to support a particular hypothesis is often substantially limited. As diagnostic testing moves to include these genes in routinely available tests, there is a need for an efficient and reproducible method for evaluating the strength of the evidence suggesting a causal relationship. Meaningful panel design, and the appropriate understanding and use of results derived from the included genes, depend on this fundamental understanding.

### Clinical Validity of a Panel Test

This paper aims to provide a method for establishing clinical validity of individual genes that is consistent with the general ACCE framework. We also note that, although clinical practice is quickly moving to embrace panel testing, a clear framework does not exist for establishing clinical validity on a panel level. Some entities suggest that clinical validity of a panel is established when each and every included gene has established validity; however, this assertion is refuted by the rapid adoption of exome testing as a viable, clinically valid option.

For many conditions, the bulk of the diagnostic yield of a panel test is accounted for by pathogenic variants in a small number of genes, and is supplemented by a “long tail” of genes that account for rare cases. In addition, there can be real benefit to patients to testing genes in advance of their clinical validity being conclusively established.

We therefore propose that the clinical validity of the panel test is largely established by the inclusion of genes that account for the bulk of the diagnostic yield for that condition. Conversely, a panel test should be considered to be out-of-date and no longer clinically valid if it fails to include such genes. For example, a clinically valid test for HCM must include *MYBPC3*, as pathogenic variants in this gene account for a substantial portion of HCM cases, and an HCM panel that fails to include this gene should not be. However, a panel should not be bounded by the current state of information, for the reasons described below. An effective HCM panel may also include a series of preliminary evidence genes that may turn out to contribute additional clinical sensitivity, but that cannot do so at this point in time.

### Utility of Findings in the Three Classes of Genes

Broadly speaking, genes are included in a panel for one of the three reasons listed below. The utility of findings in the gene depends on the categorization of the gene and the reason for inclusion.

#### Genes That Definitively Cause a Condition within the Patient’s Differential

When pathogenic variants are identified in these genes, these variants likely represent a causal explanation for the individual’s condition. Pathogenic variants in these genes can inform the prognosis, management, and treatment of the affected individual. Pathogenic variants are also material to the health and clinical management of the proband’s family members. Asymptomatic relatives who carry the variant may be candidates for more aggressive screening, monitoring, or prophylactic interventions. Asymptomatic relatives who do not carry the pathogenic familial variants may be returned to standard monitoring protocols for their demographic.

When variants of uncertain significance (VUSes) are identified in these genes, testing of similarly affected family members may be useful in understanding the clinical significance of the variant. Segregation of the variant with disease can inform the relevance of the variant to the particular family, and may inform the formal classification of the variant.

Most of the diagnostic yield from a panel test is derived from these genes, and testing broadly beyond this class of genes does not substantially increase that yield (35).

#### Genes That Definitively Cause a Related or Similar Condition, but That Have Not Been Definitively Proven to Cause the Proband’s Condition

The clinical overlap between many cardiac conditions is extensive. Furthermore, we know that our understanding of the full phenotypic heterogeneity of many of these genes may be limited. It will come as no surprise when evidence emerges demonstrating that pathogenic variants in any one gene can lead to a larger range of phenotypes than we currently appreciate. Because of this, more clinicians are opting to test genes that have been definitively proven to cause a related disease in their diagnostic testing regimens. However, the utility of findings in such genes is different than that described above.

When pathogenic variants are identified in these genes, they can mean one of a few things. The variant may represent the true cause of the patient’s condition, and may indicate that the patient represents an expansion of the clinical phenotype previously associated with the gene. However, due to the prevalence of some cardiac conditions, families affected by more than one condition are not uncommon. The pathogenic variant may, therefore, be an incidental finding, and may indicate that the individual is also at risk for a second condition. The pathogenic finding is still relevant to asymptomatic family members; however, caution should be applied as the observation in the first proband may suggest that the variant is incompletely penetrant in this family. Discovery of pathogenic or uncertain variants in these genes in a patient should stimulate a thorough review of the clinical presentation through the lens of the new hypothesis. The patient may have subtle features of the associated clinical condition that were not initially appreciated.

When a VUS is identified in one of these genes, segregation data can be difficult to parse. Segregation analysis depends on the co-occurrence of the variant with an associated condition. But if it is not yet certain that the gene causes the condition, individuals with that condition are not necessarily informative. If the variant does not segregate with the condition in the family, then it is certainly not likely to be the cause of disease. In this situation, clinicians and patients must wait for additional information to emerge regarding the spectrum of clinical presentations associated with clearly pathogenic variants in the gene.

#### Genes That Have Not yet Been Proven to Cause Any Condition: Only “Suggested” Gene–Condition Relationships

Genetic testing panels routinely include “candidate” or “preliminary evidence” genes (genes with no more than “suggested” relationships to any clinical condition), and for good reason. The cost of generating and holding additional patient data has become marginal, we expect our understanding of genetics to improve rapidly over the next years, and an appropriate use of this information does not increase downstream clinical cost or burden.

It is, by definition, not possible to identify pathogenic variants in genes which have not been proven to cause any condition. Variants that are identified in these genes are not used to guide monitoring or treatment decisions. They are also not used to inform risk in family members. However, variants identified in these genes are held in the patient record or by the lab so that, if and when new information becomes available, that information can become useful to the patient without having to endure the cost and time of a second genetic test. Additionally, testing these genes can help identify patients and families who may be referred to research studies to help support an expansion of our understanding of the condition and the genetics.

### Other Necessary Information for the Accurate Interpretation of Variants

This paper focuses on establishing the clinical validity of particular genes. It should be clear, however, that although clinical validity is a primary consideration, it does not encapsulate all of the relevant details one would need to accurately interpret novel sequence variants. Such details include molecular mechanism of disease, inheritance patterns associated with disease, penetrance, age-of-onset, severity of disease, the consequence of homozygous variants, relevant protein domains, the frequency of *de novo* variants, etc. The framework for rigorously establishing these qualities is beyond the scope of this paper. If it has been proven that the gene causes disease, then questions of “how?” and “by what mechanism?” become relevant.

### Other Gene–Condition Classification Efforts

Besides the approach outlined in this paper, there exist other, promising efforts to tackle this essential question of the evaluation of the strength of the purported gene–condition relationship. Among the most promising is being developed by the ClinGen Gene Curation Working Group, a part of the broader ClinGen effort (43). This working group is developing a framework for evaluating the strength of the evidence that supports a gene–condition relationship that is similarly based on the structured evaluation of underlying evidence. We expect that group’s efforts to ultimately become the authoritative source for this sort of information. However in order for that to happen, that approach must be finalized and then broadly accepted and adopted with the support of the larger clinical genomics community. Equally importantly, it must be supported and maintained by an extensive community-based curation effort that will march through the Mendeliome and the array of possibly associated conditions. For labs and clinicians working in clinical genetics now, it is simply not possible to defer patient care until these community-based resources can mature.

## Conclusion

The design of effective diagnostic tests, the clinical validity of those tests, and the effective use of the results of such tests, depends on a clear understanding of the relationship between each gene and each considered condition. This paper clearly describes a general methodology establishing clinical validity of a gene that can easily be applied across clinical areas. For an active clinical lab, the benefits of the variant-centric approach to the question of clinical validity should be evident: it allows the lab to maintain one consistent lens for assessing clinical molecular genetics, capitalizes on the variant classification method and the infrastructure to support that method, and reduces logical inconsistencies that arise from using different schema to evaluate the relationship between genetic changes and human disease.

## Author Contributions

The conceptual framework was developed by ST, KN, and JG, with input from the individuals listed in the acknowledgments. Detailed curation and evaluation of the cardiac genes was performed by JG, JT, NJ, DB, AD, BH, and LM. Research into clinical presentation and clinical overlap was done by NJ, JT, and AD. All authors contributed to the writing and editing of the text of this paper.

## Conflict of Interest Statement

The authors of this paper are employees and stockholders of Invitae Corporation, a company that provides clinical genetic testing for cardiac and other conditions.
