# Supplementary material for: Clinical Genetic Testing for the Cardiomyopathies and Arrhythmias: A Systematic Framework for Establishing Clinical Validity and Addressing Genotypic and Phenotypic Heterogeneity
Source: Front Cardiovasc Med. 2016 Jun 27;3:20. doi: 10.3389/fcvm.2016.00020 (PMC4921949; doi:10.3389/fcvm.2016.00020)
Supplement: Supplementary file 1 [file data_sheet_1.docx]

**Supplemental table 1**

Table 1. Gene–condition strengths for cardiomyopathies (with references)

|  | HCM | DCM | ARVD/C | LVNC | Overlapping cardiomyopathy syndromes |
| --- | --- | --- | --- | --- | --- |
| *MYBPC3* | Strong (Dhandapany, *et al*., 2009) | Strong (Møller, et al., 2009; Pugh, *et al*., 2014; Waldmüller, *et al*., 2011; Zeller, *et al*., 2006) |  | Suggested (Hoedemaekers, *et al*., 2010; Probst *et al*., 2011) |  |
| *MYH7* | Strong (Andersen, *et al*., 2009; Millat, *et al*., 2010; Olivotto, *et al*., 2008) | Strong (Pugh, *et al*., 2014; Waldmüller, *et al*., 2011) |  | Strong (Hoedemaekers, *et al*., 2013; Klaassen, *et al*., 2008) | Strong: Laing distal myopathy (Lamont, *et al*., 2006, 2011) |
| *PLN* | Strong (Chiu, *et al.*, 2007b; Landstrom, *et al*., 2011) | Strong (Ceholski, *et al*., 2012; Pugh, *et al*., 2014; van Rijsingen, *et al*., 2014) | Strong (Groeneweg, *et al*., 2013; van der Zwaag, *et al*., 2012; van Rijsingen, *et al*., 2014) | Emerging (Hoedemaekers, *et al*., 2010) |  |
| *TNNC1* | Strong (Chung, *et al*., 2011; Landstrom, *et al*., 2008) | Strong (Hershberger *et al*., 2010b; Lim, *et al*., 2008; Mogensen, *et al*., 2004; van Spaendonck-Zwarts, *et al*., 2010) |  |  |  |
| *TNNT2* | Strong (Harada and Potter, 2004; Moolman, *et al*., 1997; Van Driest, *et al*., 2003; Varley, *et al*., 1997; Watkins, *et al*., 1993) | Strong (Bai, *et al*., 2013; Mogensen, *et al*., 2004; Pugh, *et al*., 2014; Rani, *et al*., 2014) |  | Strong (Klaassen, *et al*., 2008; Luedde, *et al*., 2010) |  |
| *TPM1* | Strong (Redwood and Robinson, 2013; Richard, *et al*., 2003;Van Driest, *et al*., 2003) | Strong (Lakdawala, *et al*., 2012; Pugh, *et al*., 2014) |  | Suggested (Chang, *et al*., 2011; Probst *et al*., 2011) |  |
| *TNNI3* | Strong (Millat, *et al*., 2010; Olivotto, *et al*., 2008; Richard, *et al*., 2003;Van Driest, *et* al., 2003) | Strong (Boda, *et al*., 2009; Carballo, *et al*., 2009; Lakdawala, *et al*., 2012; Murakami, *et al*., 2010; Pugh, *et al*., 2014) |  | Suggested (van den Wijngaard, *et al*., 2011) |  |
| *TTN* |  | Strong (Gerull, *et al*., 2006; Herman, *et al*., 2012) |  |  | Strong: Titinopathies (Ceyhan-Birsoy, *et al*., 2013; De Cid, *et al*., 2015; Hackman *et al*., 2002;) |
| *LMNA* |  | Strong (Parks, *et al*., 2008; Pugh, *et al*., 2014) | Suggested (Kato, *et al*., 2015; Quarta *et al*., 2012) | Suggested (Hermida-Prieto, *et al*., 2004) | Strong: Laminopathies (Rodríguez and Eriksson, 2011; Sims-Williams, 2013) |
| *DSP* |  | Strong (Elliot, *et al*., 2010; Garcia-Pavia, *et al*., 2011; Pugh, *et al*., 2014) | Strong (Basso, *et al*., 2006; Pilichou, *et al*., 2006) |  | Strong: Carvajal syndrome (Williams, *et al*., 2011) |
| *RBM20* |  | Strong (Brauch, *et al*., 2009; Li, *et al*., 2010; Pugh, *et al*., 2014; Refaat, et al.., 2012 ) | Suggested (Li, *et al*., 2010) |  |  |
| *VCL* | Emerging (Mook, *et al*., 2013; Vasile, *et al*., 2006) | Strong (Olson, et al., 2002; Pugh, *et al*., 2014; Zemljic-Harpf, *et al*., 2007) |  |  |  |
| *TAZ* |  | Strong (Aradhya, *et al*., 2002; D’Adamo, *et al*., 1997; Man, *et al*., 2013; Pugh, *et al*., 2014) |  | Strong (Ichida, *et al*., 2001; Karkucinska-Wieckowska, et al., 2013; Xing, *et al*., 2006) | Strong: Barth syndrome (Jefferies, 2013) |
| *DSG2* |  | Strong (Garcia-Pavia, *et al*., 2011; Marcus, *et al*., 2013; Pilichou, *et al*., 2006; Pugh, *et al*., 2014; Rasmussen, *et al*., 2013) | Strong (Bauce, *et al*., 2005; Norman, *et al*., 2005; Yang, *et al*., 2006) |  |  |
| *DES* |  | Strong (Li, *et al*., 1999; Kostareva, et al., 2006; Pugh, *et al*., 2014; Taylor, *et al*., 2007; Tse, *et al*., 2013) | Strong (Hedberg, *et al*., 2012; van Tintelen, *et al*., 2009) |  | Strong: Desminopathy (Goldfarb, *et al*., 2008; McLaughlin, *et al*., 2013) |
| *BAG3* |  | Strong (Arimura, *et al*., 2011; Franaszczyk, et al., 2014; Norton, *et al*., 2011) |  |  | Strong: myofibrillar myopathy (Selcen, *et al*. 2009, Lee, *et al.,* 2012) |
| *SCN5A* |  | Strong (McNair, *et al*., 2011; Olson, *et al*., 2005) | Strong (Kapplinger, *et al*., 2010; McNair, *et al*., 2011; Olson, *et al*., 2005) | Suggested (Shan, *et al*., 2008) |  |
| *DMD* |  | Strong (Diegoli, *et al*., 2011) |  |  | Strong: Duchenne muscular dystrophy (Aartsma-Rus, *et al*., 2016) |
| *RAF1* |  | Strong (Dhandapany, *et al*., 2014) |  |  |  |
| *ACTC1* | Strong (Mogensen, *et al*., 1999; Olson, *et al*., 2000) | Emerging (Debold, *et al*., 2010; Lakdawala, *et al*., 2012; Olson, *et al*., 1998) |  | Strong (Monserrat, *et al*., 2007; Rodríguez-Serrano, *et al*., 2014) |  |
| *ACTN2* | Strong (Chiu, *et al*., 2007a; Girolami *et al*., 2014) | Emerging (Mohapatra, *et al*., 2003; Pugh, *et al*., 2014) | Emerging (Bagnall, *et al*., 2014) |  |  |
| *CSRP3* | Strong (Geier, *et al*., 2008) | Suggested (Hershberger, *et al*., 2008; Mohapatra, *et al*., 2003; Pugh, *et al*., 2014; Zimmerman, *et al*., 2010) |  |  |  |
| *FHL1* | Strong (Friedrich *et al*., 2012; Hartmannova, *et al*., 2013) |  |  |  |  |
| *GLA* | Strong (Adalsteinsdottir, *et al*., 2014; Lee *et al*., 2010) |  |  |  | Strong: Fabry disease (Ellaway, 2016) |
| *MYL2* | Strong (Olivotto, *et al*., 2008; Richard, *et al*., 2003; Szczesna, *et al*., 2001) |  |  |  |  |
| *MYL3* | Strong (Poetter, *et al*., 1996) |  |  |  |  |
| *PRKAG2* | Strong (Arad, *et al*., 2002; Banerjee, *et al*., 2010; Blair, *et al*., 2001) |  |  |  | Strong: glycogen storage disease (Gollob, 2003; Wolf, *et al*., 2008) |
| *HCN4* |  |  |  | Strong (Milano, *et al*., 2014; Schweizer, *et al*., 2014) |  |
| *RYR2* |  | Emerging (Bhuiyan, *et al*., 2007) | Strong (Ackerman, et al., 2011; Ohno, *et al*., 2014) | Strong (Ohno, *et al*., 2014) |  |
| *LAMP2* |  | Emerging (Boucek, *et al*., 2011) |  |  | Strong: Danon disease (Cottinet, *et al*., 2011; Sabourdy, *et al*., 2009) |
| *TTR* |  |  |  |  | Strong: transthyretin amyloidosis (Ando, *et al*., 2005; Rowczenio, *et al*., 2014) |
| *ELAC2* |  |  |  |  | Strong: combined oxidative phosphorylation deficiency (Haack, *et al.*, 2013) |
| *MTO1* |  |  |  |  | Strong: combined oxidative phosphorylation deficiency (Baruffini, *et al*., 2013; Ghezzi, *et al*., 2012) |
| *CAV3* | Suggested (Hayashi, *et al*., 2004b) |  |  |  | Strong: Caveolinopathies (Catteruccia, *et al*., 2009; Gazzerro, *et al*., 2011) |
| *ALMS1* |  |  |  |  | Strong: Alstrom syndrome (Marshall, *et al*., 2007) |
| *EMD* |  | Emerging (Vohanka, *et al*., 2001; Zhang, *et al*., 2014) |  |  | Strong: Emery-Dreifuss muscular dystrophy (Holaska and Wilson, 2006) |
| *FKRP* |  |  |  |  | Strong: muscular dystrophy (Stehlíková, *et al*., 2014) |
| *FKTN* |  |  |  |  | Strong: muscular dystrophy (Puckett, *et al*., 2009) |
| *JUP* |  | Suggested (Garcia-Pavia, *et al*., 2011) | Strong (Asimaki, *et al*., 2007) |  | Strong: Naxos disease (Antoniades, *et al*., 2006) |
| *SDHA* |  |  |  |  | Strong: mitochondrial complex II deficiency (Levitas, *et al*., 2010; Van Coster, *et al*., 2003) |
| *TMEM43* |  |  | Strong (Merner, *et al*., 2008) |  | Suggested: Emery-Dreifuss muscular dystrophy (Liang, *et al*., 2011) |
| *DSC2* |  | Suggested (Al-Jassar, *et al*., 2013; Elliot, *et al*., 2010; Garcia-Pavia, *et al*., 2011; ) | Strong (Gerull, *et al*., 2004) |  | Strong: ARVC with palmoplantar keratoderma and woolly hair (Simpson, *et al*., 2009) |
| *PKP2* |  | Suggested (Al-Jassar, et al., 2013; Elliot, *et al*., 2010; Garcia-Pavia, *et al*., 2011; ) | Strong (Syrris, *et al*., 2006) |  |  |
| *TCAP* | Suggested (Andersen, *et al*., 2009; Hayashi, *et al*., 2004a) | Suggested (Hayashi, *et al*., 2004a; Hirtle-Lewis, *et al*., 2013 ) |  |  | Strong: Limb girdle muscular dystrophy (Moreira, *et al*., 2000) |
| *ABCC9* |  | Suggested (Bienengraeber, *et al*., 2004; Pugh, *et al*., 2014) |  |  | Strong: Cantu syndrome (Harakalova, *et al*., 2012) |
| *SGCD* |  | Suggested (Kärkkäinen, *et al*., 2003; Rutschow, *et al*., 2014; Tsubata, *et al*., 2000) |  |  | Strong: Limb girdle muscular dystrophy (Nigro, *et al*., 1996; Vainzof, *et al*., 1999) |
| The following genes have only “suggested” relationships to cardiac conditions, and are therefore classified as preliminary evidence genes: *LDB3* (Arimura, *et al*., 2009b; Banerjee, *et al*., 2010; Fratev, *et al*., 2014; Hershberger, *et al*., 2010a; Lopez-Ayala, *et al*., 2015; Vatta, *et al*., 2003; Xing, *et al*., 2006 ), *ANKRD1* (Arimura, *et al*., 2009a; Crocini, *et al*., 2013; Duboscq-Bidot, *et al*., 2009; Moulik, *et al*., 2009), *PDLIM3* (Arola, *et al*., 2007; Bagnall, *et al*., 2010; Pashmforoush, *et al*., 2001; ), *MYPN* (Duboscq-Bidot, *et a*l., 2008; Meyer, *et al*., 2013; Purevjav, *et al*., 2012), *NEXN* (Hassel, *et al*., 2009; Pugh, *et al*., 2014; Wang, *et al*., 2010), *CALR3* (Chiu, *et al.*, 2007b), JPH2 (Landstrom, *et al*., 2007), *MYLK2* (Davis, *et al*., 2001), *MYOM1* (Siegert, *et al*., 2011), *MYOZ2* (Osio, *et al*., 2007; Ruggiero, *et al*., 2013), *PRDM16* (Arndt, *et al*., 2013), *CRYAB* (Inagaki, *et al*., 2006; Pilotto, *et al*., 2006), *CTF1* (Erdmann, *et al*., 2000), *FHL2* (Arimura, *et al*., 2007), *GATA6* (Xu, *et al*., 2014), *GATAD1* (Theis, *et al*., 2011), *ILK* (Knöll, *et al*., 2007; Meder, *et al*., 2011), *LAMA4* (Knöll, *et al*., 2007), *NEBL* (Maiellaro-Rafferty, *et al*., 2013; Purevjav, *et al*., 2010), *NPPA* (Disertori, *et al*., 2013), *TMPO* (Gotic, *et al*., 2010; Taylor, *et al*., 2005), *TXNRD2* (Sibbing, *et al*., 2011), *DTNA* (Ichida, *et al*., 2001), *CTNNA3* (van Hengel, *et al*., 2013). | | | | | |

**Supplemental table 2**

Table 2. Gene–condition strengths for the arrhythmias (with references)

|  | Long QT syndrome | Short QT syndrome | Brugada syndrome | CPVT* | Overlapping arrhythmia syndrome |
| --- | --- | --- | --- | --- | --- |
| *KCNH2* | Strong (Hedley, *et al*., 2009) | Strong (Giustetto, *et al*., 2006) | Suggested (Wang, *et al*., 2014) |  |  |
| *KCNQ1* | Strong (Hedley, *et al*., 2009) | Emerging (Bellocq, *et al*., 2004; Hong, *et al*., 2005) |  |  | Strong: JLNS (Mizusawa, *et al*., 2014; Nakano, *et al*., 2016) |
| *SCN5A* | Strong (Hedley, *et al*., 2009) |  | Strong (Kapplinger, *et al*., 2010) |  |  |
| *CASQ2* |  |  |  | Strong (Faggioni and Knollmann, 2012) |  |
| *RYR2* |  |  |  | Strong (Blayney and Lai, 2009; Priori and Chen, 2011) |  |
| *CAV3* | Strong (Hedley, *et al*., 2009; Vatta, *et al*., 2006) |  |  |  |  |
| *KCNE1* | Strong (Hedley, *et al*., 2009) |  |  |  | Strong: JLNS (Mizusawa, *et al*., 2014; Nakano, *et al*., 2016) |
| *KCNE2* | Strong (Hedley, *et al*., 2009) |  |  |  |  |
| *CALM1* | Strong (Crotti, *et al*., 2013) |  |  | Strong (Crotti, *et al*., 2013; Jabbari, *et al*., 2013; Nyegaard, *et al*., 2012) |  |
| *CALM2* | Strong (Crotti, *et al*., 2013; Makita, *et al*., 2014) |  |  | Strong (Crotti, *et al*., 2013; Schwartz, *et al*., 2013) |  |
| *CALM3* | Strong (Reed, *et al*., 2015) |  |  | Strong (Crotti, *et al*., 2013; Jabbari, *et al*., 2013; Nyegaard, *et al*., 2012) |  |
| *TRDN* | Strong (Altmann, *et al*., 2015) |  |  | Strong (Napolitano, *et al*., 2004; Roux-Buisson, *et al.,* 2012) |  |
| *KCNJ2* | Emerging (Fodstad, *et al*., 2004; Lieve, *et al*., 2013) | Strong (Hattori, *et al*., 2012; Ishihara and Yan, 2007; Liu, *et al*., 2012; Priori, *et al*., 2005) |  | Emerging (Barajas-Martinez, *et al*., 2011; Jabbari, *et al*., 2013; Vega, *et al*., 2009) | Strong: Anderson-Tawil syndrome (Kostera-Pruszczyk, *et al*., 2015; Nguyen, *et al*., 2013) |
| *CACNB2* |  | Strong (Antzelevitch, *et al*., 2007) | Strong (Antzelevitch, *et al*., 2007; Burashnikov, *et al*., 2010 ) |  |  |
| *GPD1L* |  |  | Strong (London, *et al*., 2007) |  |  |
| *CACNA1C* | Emerging (Boczek, *et al*., 2013; Hedley, *et al*., 2009; Wemhöner, *et al*., 2015) | Suggested (Antzelevitch, *et al*., 2007) | Suggested (Burashnikov, *et al*., 2010; Fukuyama, *et al*., 2014) |  | Strong: Timothy syndrome (An, *et al*., 2013; Dufendach, *et al*., 2013) |
| The following genes have only “suggested” relationships to cardiac conditions, and are therefore classified as preliminary evidence genes: *SCN4B* (Hedley, *et al*., 2009; Medeiros-Domingo, *et al*., 2007), *SNTA1* (Hedley, *et al*., 2009; Wu, *et al*., 2008), *TRPM4* (Brugada, *et al*., 2005; Launay, *et al*., 2002; Liu, *et al*., 2013), *KCNE3* (Ohno, *et al*., 2011; Ravn, *et al*., 2008), *KCNE5* (Berne and Brugada, 2008; Delpón, *et al*., 2008), *RANGRF* (Brugada, *et al*., 2005; Kattygnarath, *et al*., 2011), SLMAP (Brugada, *et al*., 2005; Ishikawa, *et al*., 2012), *KCNJ8* (Barajas-Martínez, *et al*., 2012; Veeramah, *et al*., 2014), *SCN3B* (Hu, *et al*., 2009; Ishikawa, *et al*., 2013), *SCN2B* (Riuró, *et al*., 2013), and *SCN10A* (Hu, *et al*., 2014). | | | | | |

**Supplementary table references**

Aartsma-Rus, A., Ginjaar, I. B., Bushby, K. (2016). The importance of genetic diagnosis for Duchenne muscular dystrophy. *J. Med. Genet.* pii: jmedgenet-2015-103387. doi: 10.1136/jmedgenet-2015-103387

Ackerman, M. J., Priori, S. G., Willems, S., Berul, C., Brugada, R., Calkins, H., et al. (2011). HRS/EHRA expert consensus statement on the state of genetic testing for the channelopathies and cardiomyopathies this document was developed as a partnership between the Heart Rhythm Society (HRS) and the European Heart Rhythm Association (EHRA). *Heart Rhythm.* **8**(8):1308-1339. doi: 10.1016/j.hrthm.2011.05.020

Adalsteinsdottir, B., Teekakirikul, P., Maron, B. J., Burke, M. A., Gudbjartsson, D. F., Holm, H., et al. (2014). Nationwide study on hypertrophic cardiomyopathy in Iceland: evidence of a *MYBPC3* founder mutation. *Circulation*. **130**(14):1158-1167. doi: 10.1161/CIRCULATIONAHA.114.011207

Al-Jassar, C., Bikker, H., Overduin, M., Chidgey, M. (2013). Mechanistic basis of desmosome-targeted diseases. *J. Mol. Biol.* **425**(21):4006-4022. doi: 10.1016/j.jmb.2013.07.035

Altmann, H. M., Tester, D. J., Will, M. L., Middha, S., Evans, J. M., Eckloff, B. W., et al. (2015). Homozygous/compound heterozygous triadin mutations associated with autosomal-recessive long-QT syndrome and pediatric sudden cardiac arrest: elucidation of the triadin knockout syndrome. *Circulation.* **131**(23):2051-2060. doi: 10.1161/CIRCULATIONAHA.115.015397

An, H. S., Choi, E. Y., Kwon, B. S., Kim, G. B., Bae, E. J., Noh, C. I., Choi, J. Y., et al. (2013). Sudden cardiac arrest during anesthesia in a 30-month-old boy with syndactyly: a case of genetically proven Timothy syndrome. *J. Korean Med. Sci.* **28**(5):788-791. doi: 10.3346/jkms.2013.28.5.788

Andersen, P. S., Havndrup, O., Hougs, L., Sørensen, K. M., Jensen, M., Larsen, L. A., et al. (2009). Diagnostic yield, interpretation, and clinical utility of mutation screening of sarcomere encoding genes in Danish hypertrophic cardiomyopathy patients and relatives. *Hum. Mutat.* **30**(3):363-370. doi: 10.1002/humu.20862

Ando, Y., Nakamura, M., Araki, S. (2005). Transthyretin-related familial amyloidotic polyneuropathy. *Arch Neurol.* 62(7), 1057-1062.

Antoniades, L., Tsatsopoulou, A., Anastasakis, A., Syrris, P., Asimaki, A., Panagiotakos, D., et al. (2006). Arrhythmogenic right ventricular cardiomyopathy caused by deletions in plakophilin-2 and plakoglobin (Naxos disease) in families from Greece and Cyprus: genotype-phenotype relations, diagnostic features and prognosis. *Eur. Heart J.* 27(18), 2208-2216.

Antzelevitch, C., Pollevick, G. D., Cordeiro, J. M., Casis, O., Sanguinetti, M. C., Aizawa, Y., et al. (2007). Loss-of-function mutations in the cardiac calcium channel underlie a new clinical entity characterized by ST-segment elevation, short QT intervals, and sudden cardiac death. *Circulation.* 115(4), 442-449.

Arad, M., Benson, D. W., Perez-Atayde, A. R., McKenna, W. J., Sparks, E. A., Kanter, R. J., et al. (2002). Constitutively active AMP kinase mutations cause glycogen storage disease mimicking hypertrophic cardiomyopathy. *J. Clin. Invest.* 109(3), 357-362.

Aradhya, S., Lewis, R., Bonaga, T., Nwokekeh, N., Stafford, A., Boggs, B., et al. (2012). Exon-level array CGH in a large clinical cohort demonstrates increased sensitivity of diagnostic testing for Mendelian disorders. *Genet. Med.* **14**(6):594-603. doi: 10.1038/gim.2011.65

Arimura, T., Hayashi, T., Matsumoto, Y., Shibata, H., Hiroi, S., Nakamura, T., et al. (2007). Structural analysis of four and half LIM protein-2 in dilated cardiomyopathy. *Biochem. Biophys. Res. Commun.* 357(1), 162-167.

Arimura, T., Bos, J. M., Sato, A., Kubo, T., Okamoto, H., Nishi, H., et al. (2009a). Cardiac ankyrin repeat protein gene (ANKRD1) mutations in hypertrophic cardiomyopathy. *J. Am. Coll. Cardiol.* **54**(4):334-342. doi: 10.1016/j.jacc.2008.12.082

Arimura, T., Inagaki, N., Hayashi, T., Shichi, D., Sato, A., Hinohara, K., et al. (2009b). Impaired binding of ZASP/Cypher with phosphoglucomutase 1 is associated with dilated cardiomyopathy. *Cardiovasc. Res.* **83**(1):80-88. doi: 10.1093/cvr/cvp119

Arimura, T., Ishikawa, T., Nunoda, S., Kawai, S., Kimura, A. (2011). Dilated cardiomyopathy-associated BAG3 mutations impair Z-disc assembly and enhance sensitivity to apoptosis in cardiomyocytes. *Hum. Mutat.* **32**(12):1481-1491. doi: 10.1002/humu.21603

Arndt, A. K., Schafer, S., Drenckhahn, J. D., Sabeh, M. K., Plovie, E. R., Caliebe, A., et al. (2013). Fine mapping of the 1p36 deletion syndrome identifies mutation of PRDM16 as a cause of cardiomyopathy. *Am. J. Hum. Genet.* **93**(1):67-77. doi: 10.1016/j.ajhg.2013.05.015

Arola, A. M., Sanchez, X., Murphy, R. T. Hasle, E., Li, H., Elliott, P. M. (2007). Mutations in PDLIM3 and MYOZ1 encoding myocyte Z line proteins are infrequently found in idiopathic dilated cardiomyopathy. *Mol. Genet. Metab.* 90(4), 435-440.

Asimaki, A., Syrris, P., Wichter, T., Matthias, P., Saffitz, J. E., McKenna, W. J. (2007). A novel dominant mutation in plakoglobin causes arrhythmogenic right ventricular cardiomyopathy. *Am. J. Hum. Genet.* 81(5), 964-973.

Bagnall, R. D., Yeates, L., Semsarian, C. (2010). Analysis of the Z-disc genes *PDLIM3* and *MYPN* in patients with hypertrophic cardiomyopathy. *Int. J. Cardiol.* **145**(3):601-602. doi: 10.1016/j.ijcard.2010.08.004

Bagnall, R. D., Molloy, L. K., Kalman, J. M, Semsarian, C. (2014). Exome sequencing identifies a mutation in the ACTN2 gene in a family with idiopathic ventricular fibrillation, left ventricular noncompaction, and sudden death. *BMC Med. Genet.* **15**:99. doi: 10.1186/s12881-014-0099-0

Bai, F., Caster, H. M., Pinto, J. R., Kawai, M. (2013). Analysis of the molecular pathogenesis of cardiomyopathy-causing cTnT mutants I79N, ΔE96, and ΔK210. *Biophys. J.* **104**(9):1979-1988. doi: 10.1016/j.bpj.2013.04.001

Banerjee, S. K., McGaffin, K. R., Huang, X. N., Ahmad, F. (2010). Activation of cardiac hypertrophic signaling pathways in a transgenic mouse with the human PRKAG2 Thr400Asn mutation. *Biochim. Biophys. Acta.* **1802**(2):284-291. doi: 10.1016/j.bbadis.2009.12.001

Barajas-Martinez, H., Hu, D., Ontiveros, G., Caceres, G., Desai, M., Burashnikov, E., et al. (2011). Biophysical and molecular characterization of a novel de novo KCNJ2 mutation associated with Andersen-Tawil syndrome and catecholaminergic polymorphic ventricular tachycardia mimicry. *Circ. Cardiovasc. Genet.* **4**(1):51-57. doi: 10.1161/CIRCGENETICS.110.957696

Barajas-Martínez, H., Hu, D., Ferrer, T., Onetti, C. G., Wu, Y., Burashnikov, E. (2012). Molecular genetic and functional association of Brugada and early repolarization syndromes with *S422L* missense mutation in KCNJ8. *Heart Rhythm.* **9**(4):548-555. doi: 10.1016/j.hrthm.2011.10.035

Baruffini, E., Dallabona, C., Invernizzi, F., Yarham, J. W., Melchionda, L., Blakely, E. L. (2013). *MTO1* mutations are associated with hypertrophic cardiomyopathy and lactic acidosis and cause respiratory chain deficiency in humans and yeast. *Hum Mutat.* **34**(11):1501-1509. doi: 10.1002/humu.22393

Basso, C., Czarnowska, E., Della Barbera, M., Bauce, B., Beffagna, G., Wlodarska, E. K., et al. (2006). Ultrastructural evidence of intercalated disc remodelling in arrhythmogenic right ventricular cardiomyopathy: an electron microscopy investigation on endomyocardial biopsies. *Eur. Heart J.* 27(15), 1847-1854.

Bauce, B., Basso, C., Rampazzo, A., Beffagna, G., Daliento, L., Frigo, G., et al. (2005). Clinical profile of four families with arrhythmogenic right ventricular cardiomyopathy caused by dominant desmoplakin mutations. *Eur. Heart J.* 26(16), 1666-1675.

Bellocq, C., van Ginneken, A. C., Bezzina, C. R., Alders, M., Escande, D., Mannens, M. M., et al. (2004). Mutation in the *KCNQ1* gene leading to the short QT-interval syndrome. *Circulation.* 109(20), 2394-2397.

Berne, P., and Brugada, J. (2012). Brugada syndrome 2012. *Circ. J.* **76**(7), 1563-1571.

Bhuiyan, Z. A., van den Berg, M. P., van Tintelen, J. P., Bink-Boelkens, M. T., Wiesfeld, A. C., Alders, M., et al. (2007). Expanding spectrum of human RYR2-related disease: new electrocardiographic, structural, and genetic features. *Circulation.* 116(14), 1569-1576.

Bienengraeber, M., Olson, T. M., Selivanov, V. A., Kathmann, E. C., O’Cochlain, F., Gao, F. (2004). ABCC9 mutations identified in human dilated cardiomyopathy disrupt catalytic KATP channel gating. *Nat. Genet.* 36(4), 382-387.

Blair, E., Redwood, C., Ashrafian, H., Oliveira, M., Broxholme, J., Kerr, B., et al. (2001). Mutations in the gamma(2) subunit of AMP-activated protein kinase cause familial hypertrophic cardiomyopathy: evidence for the central role of energy compromise in disease pathogenesis. *Hum. Mol. Genet.* 10(11), 1215-1220.

Blayney, L. M., and Lai, F. A. (2009). Ryanodine receptor-mediated arrhythmias and sudden cardiac death. *Pharmacol. Ther.* **123**(2):151-177. doi: 10.1016/j.pharmthera.2009.03.006

Boda, U., Vadapalli, S., Calambur, N., Nallari, P. (2009). Novel mutations in beta-myosin heavy chain, actin and troponin-I genes associated with dilated cardiomyopathy in Indian population. J. Genet. 88(3), 373-377.

Boczek, N. J., Best, J. M., Tester, D. J., Giudicessi, J. R., Middha, S., Evans, J. M., et al. (2013). Exome sequencing and systems biology converge to identify novel mutations in the L-type calcium channel, *CACNA1C*, linked to autosomal dominant long QT syndrome. *Circ. Cardiovasc. Genet.* 6(3), 279-289.

Boucek, D, Jirikowic, J., Taylor, M. (2011). Natural history of Danon disease. *Genet Med.* **13**(6):563-568. doi: 10.1097/GIM.0b013e31820ad795

Brauch, K. M., Karst, M. L., Herron, K. J., de Andrade, M., Pellikka, P. A., Rodeheffer, R. J., et al. (2009). Mutations in ribonucleic acid binding protein gene cause familial dilated cardiomyopathy. *J. Am. Coll. Cardiol.* **54**(10):930-941. doi: 10.1016/j.jacc.2009.05.038

Brugada, R., Campuzano, O., Brugada, P., Brugada, J., Hong, K. (2005, updated 2014). “Brugada syndrome,” in *GeneReviews*, eds. R. A. Pagon, M. P. Adam, H. H. Ardinger, S. E. Wallace, A. Amemiya, L. J. H. Bean, et al. (Seattle: University of Washington). http://www.ncbi.nlm.nih.gov/books/NBK1517/

Burashnikov, E., Pfeiffer, R., Barajas-Martinez, H., Delpón, E., Hu, D., Desai, M., et al. (2010). Mutations in the cardiac L-type calcium channel associated with inherited J-wave syndromes and sudden cardiac death. *Heart Rhythm.* **7**(12):1872-1882. doi: 10.1016/j.hrthm.2010.08.026

Carballo, S., Robinson, P., Otway, R., Fatkin, D., Jongbloed, J. D., de Jonge, N., et al. (2009). Identification and functional characterization of cardiac troponin I as a novel disease gene in autosomal dominant dilated cardiomyopathy. *Circ. Res.* **105**(4):375-382. doi: 10.1161/CIRCRESAHA.109.196055

Catteruccia, M., Sanna, T., Santorelli, F. M., Tessa, A., Di Giacopo, R., Sauchelli, D., et al. (2009). Rippling muscle disease and cardiomyopathy associated with a mutation in the *CAV3* gene. *Neuromuscul. Disord.* **19**(11):779-783. doi: 10.1016/j.nmd.2009.08.015

Ceholski, D. K., Trieber, C. A., Holmes, C. F., Young, H.S. (2012). Lethal, hereditary mutants of phospholamban elude phosphorylation by protein kinase A. *J. Biol. Chem.* **287**(32):26596-26605. doi: 10.1074/jbc.M112.382713

Ceyhan-Birsoy, O., Agrawal, P. B., Hidalgo, C., Schmitz-Abe, K., DeChene, E. T., Swanson, L. C., et al. (2013). Recessive truncating titin gene, *TTN*, mutations presenting as centronuclear myopathy. *Neurology*. **81**(14):1205-1214. doi: 10.1212/WNL.0b013e3182a6ca62

Chang, B., Nishizawa, T., Furutani, M., Fujiki, A., Tani, M., Kawaguchi, M., et al. (2011). Identification of a novel *TPM1* mutation in a family with left ventricular noncompaction and sudden death. *Mol. Genet. Metab.* **102**(2):200-206. doi: 10.1016/j.ymgme.2010.09.009

Chiu, C., Bagnall, R. D., Ingles, J., Yeates, L., Kennerson, M., Donald, J. A., et al. (2007a). Mutations in alpha-actinin-2 cause hypertrophic cardiomyopathy: a genome-wide analysis. *J. Am. Coll. Cardiol.* **55**(11):1127-1135. doi: 10.1016/j.jacc.2009.11.016

Chiu, C., Tebo, M., Ingles, J., Yeates, L., Arthur, J. W., Lind, J. M., et al. (2007b). Genetic screening of calcium regulation genes in familial hypertrophic cardiomyopathy. *J. Mol. Cell Cardiol.* 43(3), 337-343.

Chung, W. K., Kitner, C., Maron, B. J. (2011). Novel frameshift mutation in Troponin C (*TNNC1*) associated with hypertrophic cardiomyopathy and sudden death. *Cardiol. Young.* **21**(3):345-348. doi: 10.1017/S1047951110001927

Cottinet, S. L., Bergemer-Fouquet, A. M., Toutain, A., Sabourdy, F., Maakaroun-Vermesse, Z., Levade, T., et al. (2011). Danon disease: intrafamilial phenotypic variability related to a novel LAMP-2 mutation. *J. Inherit. Metab. Dis.* **34**(2):515-522. doi: 10.1007/s10545-010-9251-y

Crocini, C., Arimura, T., Reischmann, S., Eder, A., Braren, I., Hansen, A., et al. (2013). Impact of *ANKRD1* mutations associated with hypertrophic cardiomyopathy on contraction parameters of engineered heart tissue. *Basic Res. Cardiol.* **108**(3):349. doi: 10.1007/s00395-013-0349-x

Crotti, L., Johnson, C. N., Graf, E., De Ferrari, G. M., Cuneo, B. F., Ovadia, M., et al. (2013). Calmodulin mutations associated with recurrent cardiac arrest in infants. *Circulation*. **127**(9):1009-1017. doi: 10.1161/CIRCULATIONAHA.112.001216

D’Adamo, P., Fassone, L., Gedeon, A., Janssen, E. A., Bione, S. , Bolhuis, P. A., et al. (1997). The X-linked gene G4.5 is responsible for different infantile dilated cardiomyopathies. *Am. J. Hum. Genet.* 61(4), 862-867.

Davis, J. S., Hassanzadeh, S., Winitsky, S., Lin, H., Satorius, C., Vemuri, R., et al. (2001). The overall pattern of cardiac contraction depends on a spatial gradient of myosin regulatory light chain phosphorylation. *Cell.* 107(5), 631-641.

De Cid, R., Ben Yaou, R., Roudaut, C., Charton, K., Baulande, S., Leturcq, F., et al. (2015). A new titinopathy: Childhood-juvenile onset Emery-Dreifuss-like phenotype without cardiomyopathy. *Neurology*. **85**(24):2126-2135. doi: 10.1212/WNL.0000000000002200

Debold, E. P., Saber, W., Cheema, Y., Bookwalter, C. S., Trybus, K. M., Warshaw, D. M., et al. (2010). Human actin mutations associated with hypertrophic and dilated cardiomyopathies demonstrate distinct thin filament regulatory properties *in vitro*. *J. Mol. Cell Cardiol.* **48**(2):286-292. doi: 10.1016/j.yjmcc.2009.09.014

Delpón, E., Cordeiro, J. M., Núñez, L., Thomsen, P. E., Guerchicoff, A., Pollevick, G. D., et al. (2008). Functional effects of KCNE3 mutation and its role in the development of Brugada syndrome. *Circ. Arrhythm. Electrophysiol.* **1**(3):209-218. doi: 10.1161/CIRCEP.107.748103

Dhandapany, P. S., Sadayappan, S., Xue, Y., Powell, G. T., Rani, D. S., Nallari, P., et al. (2009). A common *MYBPC3* (cardiac myosin binding protein C) variant associated with cardiomyopathies in South Asia. *Nat. Genet.* Feb;41(2):187-191. doi: 10.1038/ng.309

Dhandapany, P. S., Razzaque, M. A., Muthusami, U., Kunnoth, S., Edwards, J. J., Mulero-Navarro, S., et al. (2014). RAF1 mutations in childhood-onset dilated cardiomyopathy. *Nat. Genet.* **46**(6):635-639. doi: 10.1038/ng.2963

Diegoli, M., Grasso, M., Favalli, V., Serio, A., Gambarin, F. I., Klersy, C., et al. (2011). Diagnostic work-up and risk stratification in X-linked dilated cardiomyopathies caused by dystrophin defects. *J. Am. Coll. Cardiol.* **58**(9):925-934. doi: 10.1016/j.jacc.2011.01.072

Disertori, M., Quintarelli, S., Grasso, M., Pilotto, A., Narula, N., Favalli, V., et al. (2013). Autosomal recessive atrial dilated cardiomyopathy with standstill evolution associated with mutation of Natriuretic Peptide Precursor A. *Circ. Cardiovasc. Genet.* **6**(1):27-36. doi: 10.1161/CIRCGENETICS.112.963520

Duboscq-Bidot, L., Xu, P., Charron, P., Neyroud, N., Dilanian, G., Millaire, A., et al. (2008). Mutations in the Z-band protein myopalladin gene and idiopathic dilated cardiomyopathy. *Cardiovasc. Res.* 77(1), 118-125.

Duboscq-Bidot, L., Charron, P., Ruppert, V., Fauchier, L., Richter, A., Tavazzi, L., et al. (2009). Mutations in the ANKRD1 gene encoding CARP are responsible for human dilated cardiomyopathy. *Eur. Heart J.* **30**(17):2128-2136. doi: 10.1093/eurheartj/ehp225

Dufendach, K. A., Giudicessi, J. R., Boczek, N. J., Ackerman, M. J. (2013). Maternal mosaicism confounds the neonatal diagnosis of type 1 Timothy syndrome. *Pediatrics.* **131**(6):e1991-1995. doi: 10.1542/peds.2012-2941

Ellaway, C. (2016). Paediatric Fabry disease. *Transl. Pediatr.* **5**(1):37-42. doi: 10.3978/j.issn.2224-4336.2015.12.02

Elliott, P., O’Mahony, C., Syrris, P., Evans, A., Rivera Sorensen, C., Sheppard, M. N., et al. (2010). Prevalence of desmosomal protein gene mutations in patients with dilated cardiomyopathy. *Circ. Cardiovasc. Genet.* **3**(4):314-322. doi: 10.1161/CIRCGENETICS.110.937805

Erdmann, J., Hassfeld, S., Kallisch, H., Fleck, E., Regitz-Zagrose, V. (2000). Genetic variants in the promoter (g983G>T) and coding region (A92T) of the human cardiotrophin-1 gene (CTF1) in patients with dilated cardiomyopathy. *Hum. Mutat.* 16(5), 448.

Faggioni, M., and Knollmann, B. C. (2012). Calsequestrin 2 and arrhythmias. *Am. J. Physiol. Heart Circ. Physiol.* **302**(6):H1250-H1260. doi: 10.1152/ajpheart.00779.2011

Fodstad, H., Swan, H., Auberson, M., Gautschi, I., Loffing, J., Schild, L., et al. (2004). Loss-of-function mutations of the K(+) channel gene KCNJ2 constitute a rare cause of long QT syndrome. *J. Mol. Cell Cardiol.* 37(2), 593-602.

Franaszczyk, M., Bilinska, Z. T., Sobieszczańska-Małek, M., Michalak, E., Sleszycka, J., Sioma, A., et al. (2014). The *BAG3* gene variants in Polish patients with dilated cardiomyopathy: four novel mutations and a genotype-phenotype correlation. *J. Transl. Med.* **12**:192. doi: 10.1186/1479-5876-12-192

Fratev, F., Mihaylova, E., Pajeva, I. (2014). Combination of genetic screening and molecular dynamics as a useful tool for identification of disease-related mutations: ZASP PDZ domain G54S mutation case. *J. Chem. Inf. Model.* **54**(5):1524-1536. doi: 10.1021/ci5001136

Friedrich, F. W., Wilding, B. R., Reischmann, S., Crocini, C., Lang, P., Charron, P., et al. (2012). Evidence for *FHL1* as a novel disease gene for isolated hypertrophic cardiomyopathy. *Hum. Mol. Genet.* **21**(14):3237-3254. doi: 10.1093/hmg/dds157

Fukuyama, M., Ohno, S., Wang, Q., Shirayama, T., Itoh, H., Horie, M., et al. (2014). Nonsense-mediated mRNA decay due to a CACNA1C splicing mutation in a patient with Brugada syndrome. *Heart Rhythm.* **11**(4):629-634. doi: 10.1016/j.hrthm.2013.12.011

Garcia-Pavia, P., Syrris, P., Salas, C., Evans, A., Mirelis, J. G., Cobo-Marcos, M., et al. (2011). Desmosomal protein gene mutations in patients with idiopathic dilated cardiomyopathy undergoing cardiac transplantation: a clinicopathological study. *Heart*. **97**(21):1744-1752. doi: 10.1136/hrt.2011.227967

Gazzerro, E., Bonetto, A., Minetti, C. (2011). Caveolinopathies: translational implications of caveolin-3 in skeletal and cardiac muscle disorders. *Handb. Clin. Neurol.* **101**:135-142. doi: 10.1016/B978-0-08-045031-5.00010-4

Geier, C., Gehmlich, K., Ehler, E., Hassfeld, S., Perrot, A., Hayess, K., et al. (2008). Beyond the sarcomere: *CSRP3* mutations cause hypertrophic cardiomyopathy. *Hum. Mol. Genet.* **17**(18):2753-2765. doi: 10.1093/hmg/ddn160

Gerull, B., Heuser, A., Wichter, T., Paul, M., Basson, C. T., McDermott, D. A., et al. (2004). Mutations in the desmosomal protein plakophilin-2 are common in arrhythmogenic right ventricular cardiomyopathy. *Nat. Genet.* 36(11), 1162-1164.

Gerull, B., Atherton, J., Geupel, A., Sasse-Klaassen, S., Heuser, A., Frenneaux, M., et al. (2006). Identification of a novel frameshift mutation in the giant muscle filament titin in a large Australian family with dilated cardiomyopathy. *J. Mol. Med. (Berl).* 84(6), 478-483.

Ghezzi, D., Baruffini, E., Haack, T. B., Invernizzi, F., Melchionda, L., Dallabona, C., et al. (2012). Mutations of the mitochondrial-tRNA modifier *MTO1* cause hypertrophic cardiomyopathy and lactic acidosis. *Am. J. Hum. Genet.* **90**(6):1079-1087. doi: 10.1016/j.ajhg.2012.04.011

Girolami, F., Iascone, M., Tomberli, B., Bardi, S., Benelli, M., Marseglia, G., et al. (2014). Novel α-actinin 2 variant associated with familial hypertrophic cardiomyopathy and juvenile atrial arrhythmias: a massively parallel sequencing study. *Circ. Cardiovasc. Genet.* **7**(6):741-750. doi: 10.1161/CIRCGENETICS.113.000486

Giustetto, C., Di Monte, F., Wolpert, C., Borggrefe, M., Schimpf, R., Sbragia, P., et al. (2006). Short QT syndrome: clinical findings and diagnostic-therapeutic implications. *Eur. Heart J.* 27(20), 2440-2447.

Goldfarb, L. G., Olivé, M., Vicart, P., Goebel, H. H. (2008). Intermediate filament diseases: desminopathy. *Adv. Exp. Med. Biol.* 642, 131-164.

Gollob, M. H. (2003). Glycogen storage disease as a unifying mechanism of disease in the PRKAG2 cardiac syndrome. *Biochem. Soc. Trans.* 31(Pt 1), 228-231.

Gotic, I., Leschnik, M., Kolm, U., Markovic, M., Haubner, B. J., Biadasiewicz, K., et al. (2010). Lamina-associated polypeptide 2α loss impairs heart function and stress response in mice. *Circ. Res.* **106**(2):346-353. doi: 10.1161/CIRCRESAHA.109.205724

Groeneweg, J. A., van der Zwaag, P. A., Olde Nordkamp, L. R., Bikker, H., Jongbloed, J. D., Jongbloed, R., et al. (2013). Arrhythmogenic right ventricular dysplasia/cardiomyopathy according to revised 2010 task force criteria with inclusion of non-desmosomal phospholamban mutation carriers. *Am. J. Cardiol.* **112**(8):1197-1206. doi: 10.1016/j.amjcard.2013.06.017

Haack, T.B., Kopajtich, R., Freisinger, P., Wieland, T., Rorbach, J., Nicholls, T.J., Baruffini, E., Walther, A., Danhauser, K., Zimmermann, F.A., Husain, R.A, Schum, J., Mundy, H., Ferrero, I., Strom, T.M., Meitinger, T., Taylor, R.W., Minczuk, M., Mayr, J.A., Prokisch, H. (2013) *ELAC2* mutations cause a mitochondrial RNA processing defect associated with hypertrophic cardiomyopathy. *Am J Hum Genet.* 93(2):211-23. doi: 10.1016/j.ajhg.2013.06.006.

Hackman, P., Vihola, A., Haravuori, H., Marchand, S., Sarparanta, J., De Seze, J., et al. (2002). Tibial muscular dystrophy is a titinopathy caused by mutations in *TTN*, the gene encoding the giant skeletal-muscle protein titin. *Am. J. Hum. Genet.* 71(3), 492-500.

Harada, K., and Potter. J. D. (2004). Familial hypertrophic cardiomyopathy mutations from different functional regions of troponin T result in different effects on the pH and Ca2+ sensitivity of cardiac muscle contraction. *J. Biol. Chem.* 279(15), 14488-14495.

Harakalova, M., van Harssel, J. J., Terhal, P. A., van Lieshout, S., Duran, K., Renkens, I., et al. (2012). Dominant missense mutations in ABCC9 cause Cantú syndrome. *Nat Genet.* **44**(7):793-796. doi: 10.1038/ng.2324

Hartmannova, H., Kubanek, M., Sramko, M., Piherova, L., Noskova, L., Hodanova, K., et al. (2013). Isolated X-linked hypertrophic cardiomyopathy caused by a novel mutation of the four-and-a-half LIM domain 1 gene. *Circ. Cardiovasc. Genet.* **6**(6):543-51. doi: 10.1161/CIRCGENETICS.113.000245

Hassel, D., Dahme, T., Erdmann, J., Meder, B., Huge, A., Stoll, M., et al. (2009). Nexilin mutations destabilize cardiac Z-disks and lead to dilated cardiomyopathy. *Nat. Med.* **15**(11):1281-1288. doi: 10.1038/nm.2037

Hattori, T., Makiyama, T., Akao, M., Ehara, E., Ohno, S., Iguchi, M., et al. (2012). A novel gain-of-function *KCNJ2* mutation associated with short-QT syndrome impairs inward rectification of Kir2.1 currents. *Cardiovasc. Res.* **93**(4):666-673. doi: 10.1093/cvr/cvr329

Hayashi, T., Arimura, T., Itoh-Satoh, M., Ueda, K., Hohda, S., Inagaki, N., et al. (2004a). Tcap gene mutations in hypertrophic cardiomyopathy and dilated cardiomyopathy. *J. Am. Coll. Cardiol.* 44(11), 2192-2201.

Hayashi, T., Arimura, T., Ueda, K., Shibata, H., Hohda, S., Takahashi, M., et al. (2004b). Identification and functional analysis of a caveolin-3 mutation associated with familial hypertrophic cardiomyopathy. *Biochem. Biophys. Res. Commun.* 313(1), 178-184.

Hedberg, C., Melberg, A., Kuhl, A., Jenne, D., Oldfors, A. (2012). Autosomal dominant myofibrillar myopathy with arrhythmogenic right ventricular cardiomyopathy 7 is caused by a DES mutation. *Eur. J. Hum. Genet.* **20**(9):984-985. doi: 10.1038/ejhg.2012.39

Hedley, P. L., Jørgensen, P., Schlamowitz, S., Wangari, R., Moolman-Smook, J., Brink, P. A., et al. (2009). The genetic basis of long QT and short QT syndromes: a mutation update. *Hum. Mutat.* **30**(11):1486-1511. doi: 10.1002/humu.21106

Herman, D. S., Lam, L., Taylor, M. R., Wang, L., Teekakirikul, P., Christodoulou, D., et al. (2012). Truncations of titin causing dilated cardiomyopathy. *N. Engl. J. Med.* **366**(7):619-628. doi: 10.1056/NEJMoa1110186

Hermida-Prieto, M., Monserrat, L., Castro-Beiras, A., Laredo, R., Soler, R., Peteiro, J., et al. (2004). Familial dilated cardiomyopathy and isolated left ventricular noncompaction associated with lamin A/C gene mutations. *Am. J. Cardiol.* 94(1), 50-54.

Hershberger, R. E., Parks, S. B., Kushner, J .D., Li, D., Ludwigsen, S., Jakobs, P., et al. (2008). Coding sequence mutations identified in *MYH7*, *TNNT2*, *SCN5A*, *CSRP3*, *LBD3*, and *TCAP* from 313 patients with familial or idiopathic dilated cardiomyopathy. *Clin. Transl. Sci.* **1**(1):21-26. doi: 10.1111/j.1752-8062.2008.00017.x

Hershberger, R. E., Morales, A., Siegfried, J. D. (2010a). Clinical and genetic issues in dilated cardiomyopathy: a review for genetics professionals. *Genet. Med.* **12**(11):655-667. doi: 10.1097/GIM.0b013e3181f2481f

Hershberger, R. E., Norton, N., Morales, A., Li, D., Siegfried, J. D., Gonzalez-Quintana, J., et al. (2010b). Coding sequence rare variants identified in MYBPC3, MYH6, TPM1, TNNC1, and TNNI3 from 312 patients with familial or idiopathic dilated cardiomyopathy. *Circ. Cardiovasc. Genet.* **3**(2):155-561. doi: 10.1161/CIRCGENETICS.109.912345

Hirtle-Lewis, M., Desbiens, K., Ruel, I., Rudzicz, N., Genest, J., Engert, J. C., et al. (2013). The genetics of dilated cardiomyopathy: a prioritized candidate gene study of LMNA, TNNT2, TCAP, and PLN. *Clin. Cardiol.* **36**(10):628-633. doi: 10.1002/clc.22193

Hoedemaekers, Y. M., Caliskan, K., Michels, M., Frohn-Mulder, I., van der Smagt, J. J., Phefferkorn, J. E., et al. (2010). The importance of genetic counseling, DNA diagnostics, and cardiologic family screening in left ventricular noncompaction cardiomyopathy. *Circ. Cardiovasc. Genet.* **3**(3):232-239. doi: 10.1161/CIRCGENETICS.109.903898

Holaska, J. M., and Wilson, K. L. (2006). Multiple roles for emerin: implications for Emery-Dreifuss muscular dystrophy. *Anat. Rec. A. Discov. Mol. Cell. Evol. Biol.* 288(7), 676-680.

Hong, K., Piper, D. R., Diaz-Valdecantos, A., Brugada, J., Oliva, A., Burashnikov, E., et al. (2005). De novo *KCNQ1* mutation responsible for atrial fibrillation and short QT syndrome in utero. *Cardiovasc. Res.* 68(3), 433-440.

Hu, D., Barajas-Martinez, H., Burashnikov, E., Springer, M., Wu, Y., Varro, A., et al. (2009). A mutation in the beta 3 subunit of the cardiac sodium channel associated with Brugada ECG phenotype. *Circ. Cardiovasc. Genet.* **2**(3):270-278. doi: 10.1161/CIRCGENETICS.108.829192

Hu, D., Barajas-Martínez, H., Pfeiffer, R., Dezi, F., Pfeiffer, J., Buch, T., et al. (2014). Mutations in *SCN10A* are responsible for a large fraction of cases of Brugada syndrome. *J. Am. Coll. Cardiol.* **64**(1):66-79. doi: 10.1016/j.jacc.2014.04.032

Ichida, F., Tsubata, S., Bowles, K. R., Haneda, N., Uese, K., Miyawaki, T., et al. (2001). Novel gene mutations in patients with left ventricular noncompaction or Barth syndrome. *Circulation.* 103(9), 1256-1263.

Inagaki, N., Hayashi, T., Arimura, T., Koga, Y., Takahashi, M., Shibata, H., et al. (2006). αβ-Crystallin mutation in dilated cardiomyopathy. *Biochem. Biophys. Res. Commun.* 342(2), 379-386.

Ishihara, K., and Yan, D. H. (2007). Low-affinity spermine block mediating outward currents through Kir2.1 and Kir2.2 inward rectifier potassium channels. *J. Physiol.* 583(Pt 3), 891-908.

Ishikawa, T., Sato, A., Marcou, C. A., Tester, D. J., Ackerman, M. J., Crotti, L., et al. (2012). A novel disease gene for Brugada syndrome: sarcolemmal membrane-associated protein gene mutations impair intracellular trafficking of hNav1.5. *Circ. Arrhythm. Electrophysiol.* **5**(6):1098-1107. doi: 10.1161/CIRCEP.111.969972

Ishikawa, T., Takahashi, N., Ohno, S., Sakurada, H., Nakamura, K., On, Y. K., et al. (2013). Novel SCN3B mutation associated with Brugada syndrome affects intracellular trafficking and function of Nav1.5. *Circ. J.* 77(4), 959-967.

Jabbari, J., Jabbari, R., Nielsen, M. W., Holst, A. G., Nielsen, J. B., Haunsø, S., et al. (2013). New exome data question the pathogenicity of genetic variants previously associated with catecholaminergic polymorphic ventricular tachycardia. *Circ. Cardiovasc. Genet.* **6**(5):481-489. doi: 10.1161/CIRCGENETICS.113.000118

Jefferies, J. L. (2013). Barth syndrome. *Am. J. Med. Genet. C. Semin. Med. Genet.* **163C**(3):198-205. doi: 10.1002/ajmg.c.31372

Kapplinger, J. D., Tester, D. J., Alders, M., Benito, B., Berthet, M., Brugada, J., et al. (2010). An international compendium of mutations in the *SCN5A*-encoded cardiac sodium channel in patients referred for Brugada syndrome genetic testing. *Heart Rhythm.* **7**(1):33-46. doi: 10.1016/j.hrthm.2009.09.069

Kärkkäinen, S., Miettinen, R., Tuomainen, P., Kärkkäinen, P., Heliö, T., Reissell, E., et al. (2003). A novel mutation, Arg71Thr, in the delta-sarcoglycan gene is associated with dilated cardiomyopathy. *J. Mol. Med. (Berl).* 81(12), 795-800.

Karkucinska-Wieckowska, A., Trubicka, J., Werner, B., Kokoszynska, K., Pajdowska, M., Pronicki, M., et al. (2013). Left ventricular noncompaction (LVNC) and low mitochondrial membrane potential are specific for Barth syndrome. *J. Inherit. Metab. Dis.* **36**(6):929-937. doi: 10.1007/s10545-013-9584-4

Kato, K., Takahashi, N., Fujii, Y., Umehara, A., Nishiuchi, S., Makiyama, T., et al. (2015). LMNA cardiomyopathy detected in Japanese arrhythmogenic right ventricular cardiomyopathy cohort. *J. Cardiol.* In press. doi: 10.1016/j.jjcc.2015.10.013

Kattygnarath, D., Maugenre, S., Neyroud, N., Balse, E., Ichai, C., Denjoy, I., et al. (2011). *MOG1*: a new susceptibility gene for Brugada syndrome. *Circ. Cardiovasc. Genet.* **4**(3):261-268. doi: 10.1161/CIRCGENETICS.110.959130

Klaassen, S., Probst, S., Oechslin, E., Gerull, B., Krings, G., Schuler, P., et al. (2008). Mutations in sarcomere protein genes in left ventricular noncompaction. *Circulation*. **117**(22):2893-2901. doi: 10.1161/CIRCULATIONAHA.107.746164

Knöll, R., Postel, R., Wang, J., Krätzner, R., Hennecke, G., Vacaru, A. M., et al. (2007). Laminin-α4 and integrin-linked kinase mutations cause human cardiomyopathy via simultaneous defects in cardiomyocytes and endothelial cells. *Circulation.* 116(5), 515-525.

Kostareva, A., Gudkova, A., Sjoberg, G., Kiselev, I., Moiseeva, O., Karelkina, E., et al. (2006). Desmin mutations in a St. Petersburg cohort of cardiomyopathies. *Acta Myol.* 25(3), 109-115.

Kostera-Pruszczyk, A., Potulska-Chromik, A., Pruszczyk, P., Bieganowska, K., Miszczak-Knecht, M., Bienias, P., et al. (2015). Andersen-Tawil syndrome: report of 3 novel mutations and high risk of symptomatic cardiac involvement. *Muscle Nerve.* **51**(2):192-196. doi: 10.1002/mus.24293.

Lakdawala, N. K., Funke, B. H., Baxter, S., Cirino, A. L., Roberts, A. E., Judge, D. P., et al. (2012). Genetic testing for dilated cardiomyopathy in clinical practice. *J. Card. Fail.* **18**(4):296-303. doi: 10.1016/j.cardfail.2012.01.013

Lamont, P. J., Udd, B., Mastaglia, F. L., de Visser, M., Hedera, P., Voit, T., et al. (2006). Laing early onset distal myopathy: slow myosin defect with variable abnormalities on muscle biopsy. *J. Neurol. Neurosurg. Psychiatry*. 77(2), 208-215.

Lamont, P., Wallefeld, W., Davis, M., Udd, B., Laing, N. (2011). Clinical utility gene card for: Laing distal myopathy. *Eur. J. Hum. Genet.* **19**(3). doi: 10.1038/ejhg.2010.190

Landstrom, A. P., Weisleder, N., Batalden, K. B., Bos, J. M., Tester, D. J., Ommen, S. R., et al. (2007). Mutations in JPH2-encoded junctophilin-2 associated with hypertrophic cardiomyopathy in humans. *J. Mol. Cell Cardiol.* 42(6), 1026-1035.

Landstrom, A. P., Parvatiyar, M. S., Pinto, J. R., Marquardt, M.L., Bos, J. M., Tester, D. J., et al. (2008), Molecular and functional characterization of novel hypertrophic cardiomyopathy susceptibility mutations in TNNC1-encoded troponin C. *J. Mol. Cell Cardiol.* **45**(2):281-288. doi: 10.1016/j.yjmcc.2008.05.003

Landstrom, A. P., Adekola, B. A., Bos, J. M., Ommen, S. R., Ackerman, M. J. (2011). *PLN*-encoded phospholamban mutation in a large cohort of hypertrophic cardiomyopathy cases: summary of the literature and implications for genetic testing. *Am. Heart J.* **161**(1):165-171. doi: 10.1016/j.ahj.2010.08.001

Launay, P., Fleig, A., Perraud, A . L., Scharenberg, A. M., Penner, R., Kinet, J. P. (2002). TRPM4 is a Ca^2+^-activated nonselective cation channel mediating cell membrane depolarization. *Cell.* 109(3), 397-407.

Lee, B. H., Heo, S. H., Kim, G. H., Park, J. Y., Kim, W. S., Kang, D. H., et al. (2010). Mutations of the GLA gene in Korean patients with Fabry disease and frequency of the E66Q allele as a functional variant in Korean newborns. J. Hum. Genet. 55(8):512-517. doi: 10.1038/jhg.2010.58

Lee H. C., Cherk S. W., Chan S. K., Wong S., Tong T. W., Ho W. S., Chan A. Y., Lee K. C., Mak C. M. (2012) *BAG3*-related myofibrillar myopathy in a Chinese family. *Clin Genet*. Apr;81(4):394-8. doi: 10.1111/j.1399-0004.2011.01659

Levitas, A., Muhammad, E., Harel, G., Saada, A., Caspi, V. C., Manor, E., et al. (2010). Familial neonatal isolated cardiomyopathy caused by a mutation in the flavoprotein subunit of succinate dehydrogenase. *Eur. J. Hum. Genet.* **18**(10):1160-1165. doi: 10.1038/ejhg.2010.83

Li, D., Tapscoft, T., Gonzalez, O., Burch, P. E., Quiñones, M. A., Zoghbi, W. A., et al. (1999). Desmin mutation responsible for idiopathic dilated cardiomyopathy. *Circulation.* 100(5), 461-464.

Li, D., Morales, A., Gonzalez-Quintana, J., Norton, N., Siegfried, J. D., Hofmeyer, M., Hershberger, R. E. (2010). Identification of novel mutations in *RBM20* in patients with dilated cardiomyopathy. *Clin. Transl. Sci.* **3**(3), 90-97. doi: 10.1111/j.1752-8062.2010.00198.x

Liang, W. C., Mitsuhashi, H., Keduka, E., Nonaka, I., Noguchi, S., Nishino, I., et al. (2011). *TMEM43* mutations in Emery-Dreifuss muscular dystrophy-related myopathy. *Ann. Neurol.* **69**(6):1005-1013. doi: 10.1002/ana.22338

Lieve, K. V., Williams, L., Daly, A., Richard, G., Bale, S., Macaya, D., Chung, W. K. (2013). Results of genetic testing in 855 consecutive unrelated patients referred for long QT syndrome in a clinical laboratory. *Genet. Test Mol. Biomarkers.* **17**(7):553-561. doi: 10.1089/gtmb.2012.0118

Lim, C. C., Yang, H., Yang, M., Wang, C. K., Shi, J., Berg, E. A., et al. (2008). A novel mutant cardiac troponin C disrupts molecular motions critical for calcium binding affinity and cardiomyocyte contractility. *Biophys. J.* **94**(9):3577-3589. doi: 10.1529/biophysj.107.112896

Liu, H., Chatel, S., Simard, C., Syam, N., Salle, L., Probst, V., et al. (2013). Molecular genetics and functional anomalies in a series of 248 Brugada cases with 11 mutations in the TRPM4 channel. *PLoS One.* 2013;**8**(1):e54131. doi: 10.1371/journal.pone.0054131

Liu, T. A., Chang, H. K., Shieh, R. C. (2012). Revisiting inward rectification: K ions permeate through Kir2.1 channels during high-affinity block by spermidine. *J. Gen. Physiol.* **139**(3):245-259. doi: 10.1085/jgp.201110736

London, B., Michalec, M., Mehdi, H., Zhu, X., Kerchner, L., Sanyal, S., et al. (2007). Mutation in glycerol-3-phosphate dehydrogenase 1 like gene (*GPD1-L*) decreases cardiac Na+ current and causes inherited arrhythmias. *Circulation.* 116(20), 2260-2268.

Lopez-Ayala, J. M., Ortiz-Genga, M., Gomez-Milanes, I., Lopez-Cuenca, D., Ruiz-Espejo, F., Sanchez-Munoz, J. J., et al. (2015). A mutation in the Z-line Cypher/ZASP protein is associated with arrhythmogenic right ventricular cardiomyopathy. *Clin. Genet.* **88**(2):172-176. doi: 10.1111/cge.12458

Luedde, M., Ehlermann, P., Weichenhan, D., Will, R., Zeller, R., Rupp, S., et al. (2010). Severe familial left ventricular non-compaction cardiomyopathy due to a novel troponin T (*TNNT2*) mutation. *Cardiovasc. Res.* **86**(3):452-460. doi: 10.1093/cvr/cvq009

Maiellaro-Rafferty, K., Wansapura, J. P., Mendsaikhan, U., Osinska, H., James, J. F., Taylor, M. D. (2013). Altered regional cardiac wall mechanics are associated with differential cardiomyocyte calcium handling due to nebulette mutations in preclinical inherited dilated cardiomyopathy. *J. Mol. Cell Cardiol.* **60**:151-160. doi: 10.1016/j.yjmcc.2013.04.021

Makita, N., Yagihara, N., Crotti. L., Johnson, C. N., Beckmann, B. M., Roh, M. S., et al. (2014). Novel calmodulin mutations associated with congenital arrhythmia susceptibility. *Circ. Cardiovasc. Genet.* **7**(4):466-474. doi: 10.1161/CIRCGENETICS.113.000459

Man, E., Lafferty, K. A., Funke, B. H., Lun, K. S., Chan, S. Y., Chau, A. K., et al. (2013). NGS identifies *TAZ* mutation in a family with X-linked dilated cardiomyopathy. *BMJ Case Rep.* **2013**. pii: bcr2012007529. doi: 10.1136/bcr-2012-007529

Marcus, F. I., Edson, S., Towbin, J. A. (2013). Genetics of arrhythmogenic right ventricular cardiomyopathy: a practical guide for physicians. *J. Am. Coll. Cardiol.* **61**(19):1945-1948. doi: 10.1016/j.jacc.2013.01.073

Marshall, J. D., Hinman, E. G., Collin, G. B., Beck, S., Cerqueira, R., Maffei, P., et al. (2007). Spectrum of *ALMS1* variants and evaluation of genotype-phenotype correlations in Alström syndrome. Hum. Mutat. 28(11):1114-1123.

McLaughlin, H. M., Kelly, M. A., Hawley, P. P., Darras, B. T., Funke, B., Picker, J. (2013). Compound heterozygosity of predicted loss-of-function DES variants in a family with recessive desminopathy. *BMC Med. Genet.* **14**:68. doi: 10.1186/1471-2350-14-68.

McNair, W. P., Sinagra, G., Taylor, M. R., Di Lenarda, A., Ferguson, D. A., Salcedo, E. E., et al. (2011). *SCN5A* mutations associate with arrhythmic dilated cardiomyopathy and commonly localize to the voltage-sensing mechanism. *J. Am. Coll. Cardiol.* **57**(21):2160-2168. doi: 10.1016/j.jacc.2010.09.084

Medeiros-Domingo, A., Kaku, T., Tester, D. J., Iturralde-Torres, P., Itty, A., Ye, B., et al. (2007). *SCN4B*-encoded sodium channel beta4 subunit in congenital long-QT syndrome. *Circulation.* 116(2), 134-142.

Meder, B., Haas, J., Keller, A., Heid, C., Just, S., Borries, A., et al. (2011). Targeted next-generation sequencing for the molecular genetic diagnostics of cardiomyopathies. *Circ. Cardiovasc. Genet.* **4**(2):110-122. doi: 10.1161/CIRCGENETICS.110.958322

Merner, N. D., Hodgkinson, K. A., Haywood, A. F., Connors, S., French, V. M., Drenckhahn, J. D., et al. (2008). Arrhythmogenic right ventricular cardiomyopathy type 5 is a fully penetrant, lethal arrhythmic disorder caused by a missense mutation in the *TMEM43* gene. *Am. J. Hum. Genet.* **82**(4):809-821. doi: 10.1016/j.ajhg.2008.01.010

Meyer, T., Ruppert, V., Ackermann, S., Richter, A., Perrot, A., Sperling, S. R., et al. (2013). Novel mutations in the sarcomeric protein myopalladin in patients with dilated cardiomyopathy. *Eur. J. Hum. Genet.* **21**(3):294-300. doi: 10.1038/ejhg.2012.173

Milano, A., Vermeer, A. M., Lodder, E. M., Barc, J., Verkerk, A. O., Postma, A.V., et al. (2014). *HCN4* mutations in multiple families with bradycardia and left ventricular noncompaction cardiomyopathy. *J. Am. Coll. Cardiol.* **64**(8):745-756. doi: 10.1016/j.jacc.2014.05.045

Millat, G., Bouvagnet, P., Chevalier, P., Dauphin, C., Jouk, P. S., Da Costa, A., et al. (2010). Prevalence and spectrum of mutations in a cohort of 192 unrelated patients with hypertrophic cardiomyopathy. *Eur. J. Med. Genet.* **53**(5):261-267. doi: 10.1016/j.ejmg.2010.07.007

Mizusawa, Y., Horie, M., Wilde, A. A. (2014). Genetic and clinical advances in congenital long QT syndrome. *Circ. J.* 78(12), 2827-2833.

Mogensen, J., Klausen, I. C., Pedersen, A. K., Egeblad, H., Bross, P., Kruse, T. A., et al. (1999). Alpha-cardiac actin is a novel disease gene in familial hypertrophic cardiomyopathy. *J. Clin. Invest.* 103(10), R39-R43.

Mogensen, J., Murphy, R. T., Shaw, T., Bahl, A., Redwood, C., Watkins, H., et al. (2004). Severe disease expression of cardiac troponin C and T mutations in patients with idiopathic dilated cardiomyopathy. *J. Am. Coll. Cardiol.* 44(10), 2033-2040.

Mohapatra, B., Jimenez, S., Lin, J. H., Bowles, K. R., Coveler, K. J., Marx, J. G., et al. (2003). Mutations in the muscle LIM protein and alpha-actinin-2 genes in dilated cardiomyopathy and endocardial fibroelastosis. *Mol. Genet. Metab.* 80(1-2), 207-215.

Møller, D. V., Andersen, P. S., Hedley, P., Ersbøll, M. K., Bundgaard, H., Moolman-Smook, J., et al. (2009). The role of sarcomere gene mutations in patients with idiopathic dilated cardiomyopathy. *Eur. J. Hum. Genet.* **17**(10):1241-1249. doi: 10.1038/ejhg.2009.34

Monserrat, L., Hermida-Prieto, M., Fernandez, X., Rodríguez, I., Dumont, C., Cazón, L., et al. (2007). Mutation in the alpha-cardiac actin gene associated with apical hypertrophic cardiomyopathy, left ventricular non-compaction, and septal defects. *Eur. Heart J.* 28(16), 1953-1961

Mook, O. R., Haagmans, M. A., Soucy, J. F., van de Meerakker, J. B., Baas, F., Jakobs, M. E. (2013). Targeted sequence capture and GS-FLX Titanium sequencing of 23 hypertrophic and dilated cardiomyopathy genes: implementation into diagnostics. *J. Med. Genet.* **50**(9):614-626. doi: 10.1136/jmedgenet-2012-101231

Moolman, J. C., Corfield, V. A., Posen, B., Ngumbela, K., Seidman, C., Brink, P. A., et al. (1997). Sudden death due to troponin T mutations. *J. Am. Coll. Cardiol.* 29(3), 549-555.

Moreira, E. S., Wiltshire, T. J., Faulkner, G., Nilforoushan, A., Vainzof, M., Suzuki, O. T., et al. (2000). Limb-girdle muscular dystrophy type 2G is caused by mutations in the gene encoding the sarcomeric protein telethonin. *Nat Genet.* 24(2), 163-166.

Moulik, M., Vatta, M., Witt, S. H., Arola, A. M., Murphy, R. T., McKenna, W. J., et al. (2009). *ANKRD1*, the gene encoding cardiac ankyrin repeat protein, is a novel dilated cardiomyopathy gene. *J. Am. Coll. Cardiol.* **54**(4):325-333. doi: 10.1016/j.jacc.2009.02.076

Murakami, C., Nakamura, S., Kobayashi, M., Maeda, K., Irie, W., Wada, B., et al. (2010). Analysis of the sarcomere protein gene mutation on cardiomyopathy - Mutations in the cardiac troponin I gene. *Leg. Med. (Tokyo).* **12**(6):280-283. doi: 10.1016/j.legalmed.2010.07.002

Nakano, Y., and Shimizu, W. (2016). Genetics of long-QT syndrome. *J. Hum. Genet.* **61**(1):51-55. doi: 10.1038/jhg.2015.74

Napolitano, C., Priori, S. G., Bloise, R. (2004). “Catecholaminergic polymorphic ventricular tachycardia,” in *GeneReviews*, eds. R. A. Pagon, M. P. Adam, H. H. Ardinger, S. E. Wallace, A. Amemiya, L. J. H. Bean, T. D., et al. (Seattle: University of Washington). http://www.ncbi.nlm.nih.gov/books/NBK1289/

Nguyen, H. L., Pieper, G. H., Wilders, R. (2013). Andersen-Tawil syndrome: clinical and molecular aspects. *Int. J. Cardiol.* 170(1), 1-16.

Nigro, V., de Sá Moreira, E., Piluso, G., Vainzof, M., Belsito, A., Politano, L., et al. (1996). Autosomal recessive limb-girdle muscular dystrophy, LGMD2F, is caused by a mutation in the delta-sarcoglycan gene. *Nat. Genet.* 14(2), 195-198.

Norman, M., Simpson, M., Mogensen, J., Shaw, A., Hughes, S., Syrris, P., et al. (2005).

Novel mutation in desmoplakin causes arrhythmogenic left ventricular cardiomyopathy. *Circulation.* 112(5), 636-642.

Norton, N., Li, D., Rieder, M. J., Siegfried, J. D., Rampersaud, E., Züchner, S., et al. (2011). Genome-wide studies of copy number variation and exome sequencing identify rare variants in BAG3 as a cause of dilated cardiomyopathy. *Am. J. Hum. Genet.* **88**(3):273-282. doi: 10.1016/j.ajhg.2011.01.016

Nyegaard, M., Overgaard, M. T., Søndergaard, M. T., Vranas, M., Behr, E. R., Hildebrandt, L., et al. (2012). Mutations in calmodulin cause ventricular tachycardia and sudden cardiac death. *Am. J. Hum. Genet.* **91**(4):703-712. doi: 10.1016/j.ajhg.2012.08.015

Ohno, S., Zankov, D. P., Ding, W. G., Itoh, H., Makiyama, T., Doi, T., et al. (2011). KCNE5 (KCNE1L) variants are novel modulators of Brugada syndrome and idiopathic ventricular fibrillation. *Circ. Arrhythm. Electrophysiol.* **4**(3):352-361. doi: 10.1161/CIRCEP.110.959619

Ohno, S., Omura, M., Kawamura, M., Kimura, H., Itoh, H., Makiyama, T., et al. (2014). Exon 3 deletion of RYR2 encoding cardiac ryanodine receptor is associated with left ventricular non-compaction. *Europace.* **16**(11):1646-1654. doi: 10.1093/europace/eut382

Olivotto I, Girolami F, Ackerman MJ, Nistri S, Bos JM, Zachara E, et al. (2008). Myofilament protein gene mutation screening and outcome of patients with hypertrophic cardiomyopathy. *Mayo. Clin. Proc.* **83**(6):630-638. doi: 10.4065/83.6.630

Olson, T. M., Michels, V. V., Thibodeau, S. N., Tai, Y. S., Keating, M. T. (1998). Actin mutations in dilated cardiomyopathy, a heritable form of heart failure. *Science.* 280(5364), 750-752.

Olson, T. M., Doan, T. P., Kishimoto, N. Y., Whitby, F. G., Ackerman, M. J., Fananapazir, L. (2000). Inherited and de novo mutations in the cardiac actin gene cause hypertrophic cardiomyopathy. *J. Mol. Cell Cardiol.* 32(9), 1687-1694.

Olson, T. M., Illenberger, S., Kishimoto, N. Y., Huttelmaier, S., Keating, M. T., Jockusch, B. M. (2002). Metavinculin mutations alter actin interaction in dilated cardiomyopathy. *Circulation.* 105(4), 431-437.

Olson, T. M., Michels, V. V., Ballew, J. D., Reyna, S. P., Karst, M. L., Herron, K. J., et al. (2005). Sodium channel mutations and susceptibility to heart failure and atrial fibrillation. *JAMA.* 293(4), 447-454.

Osio, A., Tan, L., Chen, S. N., Lombardi, R., Nagueh, S. F., Shete, S., et al. (2007). Myozenin 2 is a novel gene for human hypertrophic cardiomyopathy. *Circ. Res.* 100(6), 766-768.

Parks, S. B., Kushner, J.D., Nauman, D., Burgess, D., Ludwigsen, S., Peterson, A., et al. (2008). Lamin A/C mutation analysis in a cohort of 324 unrelated patients with idiopathic or familial dilated cardiomyopathy. *Am. Heart. J.* **156**(1):161-169. doi: 10.1016/j.ahj.2008.01.026

Pashmforoush, M., Pomiès, P., Peterson, K. L., Kubalak, S., Ross, J. Jr, Hefti, A. (2001). Adult mice deficient in actinin-associated LIM-domain protein reveal a developmental pathway for right ventricular cardiomyopathy. *Nat. Med.* 7(5), 591-597.

Pilichou, K., Nava, A., Basso, C., Beffagna, G., Bauce, B., Lorenzon. A., et al. (2006). Mutations in desmoglein-2 gene are associated with arrhythmogenic right ventricular cardiomyopathy. *Circulation*. 113(9), 1171-1179.

Pilotto, A., Marziliano, N., Pasotti, M., Grasso, M., Costante, A. M., Arbustini, E. (2006). αβ-Crystallin mutation in dilated cardiomyopathies: low prevalence in a consecutive series of 200 unrelated probands. *Biochem. Biophys. Res. Commun.* 346(4), 1115-1117.

Poetter, K., Jiang, H., Hassanzadeh, S., Master, S. R., Chang, A., Dalakas, M. C., et al. (1996). Mutations in either the essential or regulatory light chains of myosin are associated with a rare myopathy in human heart and skeletal muscle. *Nat. Genet.* 13(1), 63-69.

Priori, S. G., and Chen, S. R. (2011). Inherited dysfunction of sarcoplasmic reticulum Ca2+ handling and arrhythmogenesis. *Circ. Res.* **108**(7):871-883. doi: 10.1161/CIRCRESAHA.110.226845

Priori, S. G., Pandit, S. V., Rivolta, I., Berenfeld, O., Ronchetti, E., Dhamoon, A., et al. (2005). A novel form of short QT syndrome (SQT3) is caused by a mutation in the *KCNJ2* gene. *Circ. Res.* 96(7), 800-807.

Probst, S., Oechslin, E., Schuler, P., Greutmann, M., Boyé, P., Knirsch, W., et al. (2011). Sarcomere gene mutations in isolated left ventricular noncompaction cardiomyopathy do not predict clinical phenotype. *Circ. Cardiovasc. Genet.* **4**(4):367-374. doi: 10.1161/CIRCGENETICS.110.959270

Puckett, R. L., Moore, S. A., Winder, T. L., Willer, T., Romansky, S. G., Covault, K. K., et al. (2009). Further evidence of Fukutin mutations as a cause of childhood onset limb-girdle muscular dystrophy without mental retardation. *Neuromuscul. Disord.* **19**(5):352-356. doi: 10.1016/j.nmd.2009.03.001

Pugh, T. J., Kelly, M, A., Gowrisankar, S., Hynes, E., Seidman, M. A., Baxter, S. M., et al. (2014). The landscape of genetic variation in dilated cardiomyopathy as surveyed by clinical DNA sequencing. *Genet. Med.* **16**(8):601-608. doi: 10.1038/gim.2013.204

Purevjav, E., Varela, J., Morgado, M., Kearney, D. L., Li, H., Taylor, M. D., et al. (2010). Nebulette mutations are associated with dilated cardiomyopathy and endocardial fibroelastosis. *J. Am. Coll. Cardiol.* **56**(18):1493-1502. doi: 10.1016/j.jacc.2010.05.045

Purevjav, E., Arimura, T., Augustin, S., Huby, A. C., Takagi, K., Nunoda, S., et al. (2012). Molecular basis for clinical heterogeneity in inherited cardiomyopathies due to myopalladin mutations. *Hum. Mol. Genet.* **21**(9):2039-2053. doi: 10.1093/hmg/dds022

Quarta, G., Syrris, P., Ashworth, M., Jenkins, S., Zuborne Alapi, K., Morgan, J., et al. (2012). Mutations in the Lamin A/C gene mimic arrhythmogenic right ventricular cardiomyopathy. *Eur. Heart. J.* **33**(9):1128-1136. doi: 10.1093/eurheartj/ehr451

Rani, D. S., Dhandapany, P. S., Nallari, P., Narasimhan, C., Thangaraj, K. (2014). A novel arginine to tryptophan (R144W) mutation in troponin T (cTnT) gene in an Indian multigenerational family with dilated cardiomyopathy (FDCM). *PLoS One.* **9**(7):e101451. doi: 10.1371/journal.pone.0101451

Rasmussen, T. B., Palmfeldt, J., Nissen, P. H., Magnoni, R., Dalager, S., Jensen, U. B., et al. (2013). Mutated desmoglein-2 proteins are incorporated into desmosomes and exhibit dominant-negative effects in arrhythmogenic right ventricular cardiomyopathy. *Hum. Mutat.* **34**(5):697-705. doi: 10.1002/humu.22289

Ravn, L. S., Aizawa, Y., Pollevick, G. D., Hofman-Bang, J., Cordeiro, J. M., Dixen, U., et al. (2008). Gain of function in IKs secondary to a mutation in KCNE5 associated with atrial fibrillation. *Heart Rhythm.* **5**(3):427-435. doi: 10.1016/j.hrthm.2007.12.019

Redwood, C., and Robinson, P. (2013). Alpha-tropomyosin mutations in inherited cardiomyopathies. *J. Muscle Res. Cell Motil.* **34**(3-4):285-294. doi: 10.1007/s10974-013-9358-5

Reed, G. J., Boczek, N. J., Etheridge, S. P., Ackerman, M. J. (2015). CALM3 mutation associated with long QT syndrome. *Heart Rhythm.* **12**(2):419-422. doi: 10.1016/j.hrthm.2014.10.035

Refaat, M. M., Lubitz, S. A., Makino, S., Islam, Z., Frangiskakis, J. M., Mehdi, H., et al. (2012). Genetic variation in the alternative splicing regulator RBM20 is associated with dilated cardiomyopathy. *Heart Rhythm.* **9**(3):390-396. doi: 10.1016/j.hrthm.2011.10.016

Richard, P., Charron, P., Carrier, L., Ledeuil, C., Cheav, T., Pichereau, C., et al. (2003). Hypertrophic cardiomyopathy: distribution of disease genes, spectrum of mutations, and implications for a molecular diagnosis strategy. *Circulation*. 107(17), 2227-2232.

Riuró, H., Beltran-Alvarez, P., Tarradas, A., Selga, E., Campuzano, O., Vergés, M., et al. (2013). A missense mutation in the sodium channel β2 subunit reveals SCN2B as a new candidate gene for Brugada syndrome. *Hum. Mutat.* **34**(7):961-966. doi: 10.1002/humu.22328

Rodríguez-Serrano, M., Domingo, D., Igual, B., Cano, A., Medina, P., Zorio, E. (2014). Familial left ventricular noncompaction associated with a novel mutation in the alpha-cardiac actin gene. *Rev. Esp. Cardiol. (Engl. Ed.).* **67**(10):857-859. doi: 10.1016/j.rec.2014.05.015

Roux-Buisson, N., Cacheux, M., Fourest-Lieuvin, A., Fauconnier, J., Brocard, J., Denjoy, I., et al. (2012). Absence of triadin, a protein of the calcium release complex, is responsible for cardiac arrhythmia with sudden death in human. *Hum. Mol. Genet.* **21**(12):2759-2767. doi: 10.1093/hmg/dds104

Rowczenio, D. M., Noor, I., Gillmore, J. D., Lachmann, H. J., Whelan, C., Hawkins, P. N., et al. (2014). Online registry for mutations in hereditary amyloidosis including nomenclature recommendations. *Hum. Mutat.* **35**(9):E2403-2412. doi: 10.1002/humu.22619

Ruggiero, A., Chen, S. N., Lombardi, R., Rodriguez, G., Marian, A. J. (2013). Pathogenesis of hypertrophic cardiomyopathy caused by myozenin 2 mutations is independent of calcineurin activity. *Cardiovasc. Res.* **97**(1):44-54. doi: 10.1093/cvr/cvs294

Rutschow, D., Bauer, R., Göhringer, C., Bekeredjian, R., Schinkel, S., Straub, V., et al. (2014). S151A δ-sarcoglycan mutation causes a mild phenotype of cardiomyopathy in mice. *Eur J Hum Genet.* **22**(1):119-125. doi: 10.1038/ejhg.2013.97

Sabourdy, F., Michelakakis, H., Anastasakis, A., Garcia, V., Mavridou, I., Nieto, M., et al. (2009). Danon disease: further clinical and molecular heterogeneity. *Muscle Nerve.* **39**(6):837-844. doi: 10.1002/mus.21252

Schwartz, P. J., Ackerman, M. J., George, A. L. Jr, Wilde, A. A. (2013). Impact of genetics on the clinical management of channelopathies. *J. Am. Coll. Cardiol.* **62**(3):169-180. doi: 10.1016/j.jacc.2013.04.044

Schweizer, P. A., Schröter, J., Greiner, S., Haas, J., Yampolsky, P., Mereles, D., et al. (2014). The symptom complex of familial sinus node dysfunction and myocardial noncompaction is associated with mutations in the HCN4 channel. *J. Am. Coll. Cardiol.* **64**(8):757-767. doi: 10.1016/j.jacc.2014.06.1155

Selcen D, Muntoni F, Burton BK, Pegoraro E, Sewry C, Bite AV, Engel AG. (2009). Mutation in *BAG3* causes severe dominant childhood muscular dystrophy. *Ann Neurol*. Jan;65(1):83-9. doi: 10.1002/ana.21553

Shan, L., Makita, N., Xing, Y., Watanabe, S., Futatani, T., Ye, F., et al. (2008). SCN5A variants in Japanese patients with left ventricular noncompaction and arrhythmia. Mol *Genet. Metab.* **93**(4):468-474.

Sibbing, D., Pfeufer, A., Perisic, T., Mannes, A. M., Fritz-Wolf, K., Unwin, S., et al. (2011). Mutations in the mitochondrial thioredoxin reductase gene TXNRD2 cause dilated cardiomyopathy. *Eur. Heart J.* **32**(9):1121-1133. doi: 10.1093/eurheartj/ehq507

Siegert, R., Perrot, A., Keller, S., Behlke, J., Michalewska-Włudarczyk, A., Wycisk, A., et al. (2011). A myomesin mutation associated with hypertrophic cardiomyopathy deteriorates dimerisation properties. *Biochem. Biophys. Res. Commun.* **405**(3):473-479. doi: 10.1016/j.bbrc.2011.01.056

Simpson, M. A., Mansour, S., Ahnood, D., Kalidas, K., Patton, M. A., McKenna, W. J., et al. (2009). Homozygous mutation of desmocollin-2 in arrhythmogenic right ventricular cardiomyopathy with mild palmoplantar keratoderma and woolly hair. *Cardiology.* **113**(1):28-34. doi: 10.1159/000165696

Stehlíková, K., Skálová, D., Zídková, J., Mrázová, L., Vondráček, P., Mazanec, R., et al. (2014). Autosomal recessive limb-girdle muscular dystrophies in the Czech Republic. *BMC Neurol.* **14**:154. doi: 10.1186/s12883-014-0154-7

Syrris, P., Ward, D., Evans, A., Asimaki, A., Gandjbakhch, E., Sen-Chowdhry, S. et al. (2006). Arrhythmogenic right ventricular dysplasia/cardiomyopathy associated with mutations in the desmosomal gene desmocollin-2. *Am. J. Hum. Genet.* 79(5), 978-984.

Szczesna, D., Ghosh, D., Li, Q., Gomes, A. V., Guzman, G., Arana C., et al. (2001). Familial hypertrophic cardiomyopathy mutations in the regulatory light chains of myosin affect their structure, Ca2+ binding, and phosphorylation. *J. Biol. Chem.* 276(10), 7086-7092.

Taylor, M. R., Slavov, D., Gajewski, A., Vlcek, S., Ku, L., Fain, P. R., et al. (2005). Thymopoietin (lamina-associated polypeptide 2) gene mutation associated with dilated cardiomyopathy. *Hum Mutat.* 26(6), 566-574.

Taylor, M. R., Slavov, D., Ku, L., Di Lenarda, A., Sinagra, G., Carniel, E., et al. (2007). Prevalence of desmin mutations in dilated cardiomyopathy. *Circulation.* 115(10), 1244-51.

Theis, J. L., Sharpe, K. M., Matsumoto, M. E., Chai, H. S., Nair, A. A., Theis, J. D., et al. (2011). Homozygosity mapping and exome sequencing reveal GATAD1 mutation in autosomal recessive dilated cardiomyopathy. *Circ. Cardiovasc. Genet.* **4**(6):585-594. doi: 10.1161/CIRCGENETICS.111.961052

Tse, H. F., Ho, J. C., Choi, S. W., Lee, Y. K., Butler, A. W., Ng, K. M., et al. (2013). Patient-specific induced-pluripotent stem cells-derived cardiomyocytes recapitulate the pathogenic phenotypes of dilated cardiomyopathy due to a novel DES mutation identified by whole exome sequencing. *Hum. Mol. Genet.* **22**(7):1395-1403. doi: 10.1093/hmg/dds556

Tsubata, S., Bowles, K. R., Vatta, M., Zintz, C., Titus, J., Muhonen, L., et al. (2000). Mutations in the human delta-sarcoglycan gene in familial and sporadic dilated cardiomyopathy. *J. Clin. Invest.* 106(5), 655-662.

Vainzof, M., Passos-Bueno, M. R., Pavanello, R. C., Marie, S. K., Oliveira, A. S., Zatz, M. (1999). Sarcoglycanopathies are responsible for 68% of severe autosomal recessive limb-girdle muscular dystrophy in the Brazilian population. *J. Neurol. Sci.* 164(1), 44-49.

Van Coster, R., Seneca, S., Smet, J., Van Hecke, R., Gerlo, E., Devreese, B., et al. (2003). Homozygous Gly555Glu mutation in the nuclear-encoded 70 kDa flavoprotein gene causes instability of the respiratory chain complex II. *Am. J. Med. Genet. A.* 120A(1), 13-18.

van den Wijngaard, A., Volders, P., Van Tintelen, J. P., Jongbloed, J. D., van den Berg, M. P., Lekanne Deprez, R. H., et al. (2011). Recurrent and founder mutations in the Netherlands: cardiac Troponin I (*TNNI3*) gene mutations as a cause of severe forms of hypertrophic and restrictive cardiomyopathy. *Neth. Heart J.* **19**(7-8):344-351. doi: 10.1007/s12471-011-0135-z

van der Zwaag, P. A., van Rijsingen, I. A., Asimaki, A., Jongbloed, J. D., van Veldhuisen, D. J., Wiesfeld, A. C., et al. (2012). Phospholamban R14del mutation in patients diagnosed with dilated cardiomyopathy or arrhythmogenic right ventricular cardiomyopathy: evidence supporting the concept of arrhythmogenic cardiomyopathy. *Eur. J. Heart Fail.* **14**(11):1199-1207. doi: 10.1093/eurjhf/hfs119

Van Driest, S. L., Ellsworth, E. G., Ommen, S. R., Tajik, A. J., Gersh, B. J., Ackerman, M. J. (2003). Prevalence and spectrum of thin filament mutations in an outpatient referral population with hypertrophic cardiomyopathy. *Circulation*. 108(4), 445-451.

van Hengel, J., Calore, M., Bauce, B., Dazzo, E., Mazzotti, E., De Bortoli, M., et al. (2013). Mutations in the area composita protein αT-catenin are associated with arrhythmogenic right ventricular cardiomyopathy. *Eur. Heart J.* **34**(3):201-210. doi: 10.1093/eurheartj/ehs373

van Rijsingen, I. A., van der Zwaag, P. A., Groeneweg, J. A., Nannenberg, E. A., Jongbloed, J. D., Zwinderman, A. H., et al. (2014). Outcome in phospholamban R14del carriers: results of a large multicentre cohort study. *Circ. Cardiovasc. Genet.* **7**(4):455-465. doi: 10.1161/CIRCGENETICS.113.000374

van Spaendonck-Zwarts, K. Y., van Tintelen, J. P., van Veldhuisen, D. J., van der Werf, R., Jongbloed, J. D., Paulus, W. J., et al. (2010). Peripartum cardiomyopathy as a part of familial dilated cardiomyopathy. *Circulation*. **121**(20):2169-2175. doi: 10.1161/CIRCULATIONAHA.109.929646

van Tintelen, J. P., Van Gelder, I. C., Asimaki, A., Suurmeijer, A. J., Wiesfeld, A. C., Jongbloed, J. D., et al. (2009). Severe cardiac phenotype with right ventricular predominance in a large cohort of patients with a single missense mutation in the DES gene. *Heart Rhythm.* **6**(11):1574-1583. doi: 10.1016/j.hrthm.2009.07.041

Varley, J. M., McGown, G., Thorncroft, M., Santibanez-Koref, M. F., Kelsey, A. M., Tricker, K. J., et al. (1997). Germ-line mutations of *TP53* in Li-Fraumeni families: an extended study of 39 families. Cancer Res. 57(15), 3245-3252.

Vasile, V. C., Ommen, S. R., Edwards, W. D., Ackerman, M. J. (2006). A missense mutation in a ubiquitously expressed protein, vinculin, confers susceptibility to hypertrophic cardiomyopathy. *Biochem. Biophys. Res. Commun.* 345(3), 998-1003.

Vatta, M., Mohapatra, B., Jimenez, S., Sanchez, X., Faulkner, G., Perles, Z., et al. (2003). Mutations in *Cypher/ZASP* in patients with dilated cardiomyopathy and left ventricular non-compaction. *J. Am. Coll. Cardiol.* 42(11), 2014-2027.

Vatta, M., Ackerman, M. J., Ye, B., Makielski, J. C., Ughanze, E. E., Taylor, E. W., et al. (2006). Mutant caveolin-3 induces persistent late sodium current and is associated with long-QT syndrome. *Circulation.* 114(20), 2104-2112.

Veeramah, K. R., Karafet, T. M., Wolf, D., Samson, R. A., Hammer, M. F. (2014). The *KCNJ8*-S422L variant previously associated with J-wave syndromes is found at an increased frequency in Ashkenazi Jews. *Eur. J. Hum. Genet.* **22**(1):94-98. doi: 10.1038/ejhg.2013.78

Vega, A. L., Tester, D. J., Ackerman, M. J., Makielski, J. C. (2009). Protein kinase A-dependent biophysical phenotype for V227F-KCNJ2 mutation in catecholaminergic polymorphic ventricular tachycardia. *Circ. Arrhythm. Electrophysiol.* **2**(5):540-547. doi: 10.1161/CIRCEP.109.872309

Vohanka, S., Vytopil, M., Bednarik, J., Lukas, Z., Kadanka, Z., Schildberger, J., et al. (2001). A mutation in the X-linked Emery-Dreifuss muscular dystrophy gene in a patient affected with conduction cardiomyopathy. *Neuromuscul. Disord.* 11(4), 411-413.

Waldmüller, S., Erdmann, J., Binner, P., Gelbrich, G., Pankuweit, S., Geier, C., et al. (2011). Novel correlations between the genotype and the phenotype of hypertrophic and dilated cardiomyopathy: results from the German Competence Network Heart Failure. *Eur. J. Heart Fail.* **13**(11):1185-1192. doi: 10.1093/eurjhf/hfr074

Wang, H., Li, Z., Wang, J., Sun, K., Cui, Q., Song, L., et al. (2010). Mutations in *NEXN*, a Z-disc gene, are associated with hypertrophic cardiomyopathy. *Am. J. Hum. Genet.* **87**(5):687-693. doi: 10.1016/j.ajhg.2010.10.002

Wang, Q., Ohno, S., Ding, W. G., Fukuyama, M., Miyamoto, A., Itoh, H., et al. (2014). Gain-of-function *KCNH2* mutations in patients with Brugada syndrome. J Cardiovasc *Electrophysiol.* **25**(5):522-530. doi: 10.1111/jce.12361

Watkins, H., MacRae, C., Thierfelder, L., Chou, Y. H., Frenneaux, M., McKenna, W., et al. (1993). A disease locus for familial hypertrophic cardiomyopathy maps to chromosome 1q3. *Nat. Genet.* 3(4), 333-337.

Wemhöner, K., Friedrich, C., Stallmeyer, B., Coffey, A. J., Grace, A., Zumhagen, S., et al. (2015). Gain-of-function mutations in the calcium channel *CACNA1C* (Cav1.2) cause non-syndromic long-QT but not Timothy syndrome. *J. Mol. Cell Cardiol.* **80**:186-195. doi: 10.1016/j.yjmcc.2015.01.002

Williams, T., Machann, W., Kühler, L., Hamm, H., Müller-Höcker, J., Zimmer, M., et al. (2011). Novel desmoplakin mutation: juvenile biventricular cardiomyopathy with left ventricular non-compaction and acantholytic palmoplantar keratoderma. *Clin. Res. Cardiol*. **100**(12):1087-1093. doi: 10.1007/s00392-011-0345-9

Wolf, C. M., Arad, M., Ahmad, F., Sanbe, A., Bernstein, S. A., Toka, O., et al. (2008). Reversibility of PRKAG2 glycogen-storage cardiomyopathy and electrophysiological manifestations. *Circulation.* 117(2), 144-154.

Wu, G., Ai, T., Kim, J. J., Mohapatra, B., Xi, Y., Li, Z., et al. (2008). α-1-Syntrophin mutation and the long-QT syndrome: a disease of sodium channel disruption. *Circ. Arrhythm. Electrophysiol.* **1**(3):193-201. doi: 10.1161/CIRCEP.108.769224

Xing, Y., Ichida, F., Matsuoka, T., Isobe, T., Ikemoto, Y., Higaki, T., et al. (2006). Genetic analysis in patients with left ventricular noncompaction and evidence for genetic heterogeneity. *Mol. Genet. Metab.* 88(1), 71-77.

Xu, L., Zhao, L., Yuan, F., Jiang, W. F., Liu, H., Li, R. G., et al. (2014). GATA6 loss-of-function mutations contribute to familial dilated cardiomyopathy. *Int. J. Mol. Med.* **34**(5):1315-1322. doi: 10.3892/ijmm.2014.1896

Yang, Z., Bowles, N. E., Scherer, S. E., Taylor, M. D., Kearney, D. L., Ge, S., et al. (2006). Desmosomal dysfunction due to mutations in desmoplakin causes arrhythmogenic right ventricular dysplasia/cardiomyopathy. *Circ. Res.* 99(6), 646-655.

Zeller, R., Ivandic, B. T., Ehlermann, P., Mücke, O., Zugck, C., Remppis, A., et al. (2006). Large-scale mutation screening in patients with dilated or hypertrophic cardiomyopathy: a pilot study using DGGE. *J. Mol. Med. (Berl).* 84(8), 682-691.

Zemljic-Harpf, A. E., Miller, J. C., Henderson, S. A., Wright, A. T., Manso, A. M., Elsherif, L., et al. (2007). Cardiac-myocyte-specific excision of the vinculin gene disrupts cellular junctions, causing sudden death or dilated cardiomyopathy. *Mol. Cell Biol.* 27(21), 7522-7537.

Zhang, M., Chen, J., Si, D., Zheng, Y., Jiao, H., Feng, Z., et al. (2014). Whole exome sequencing identifies a novel EMD mutation in a Chinese family with dilated cardiomyopathy. *BMC Med. Genet.* **15**:77. doi: 10.1186/1471-2350-15-77

Zimmerman, R. S., Cox, S., Lakdawala, N. K., Cirino, A., Mancini-DiNardo, D., Clark, E., et al. (2010). A novel custom resequencing array for dilated cardiomyopathy. *Genet. Med.* **12**(5):268-278. doi: 10.1097/GIM.0b013e3181d6f7c0
